# Supplementary material for: Conservation and divergence of related neuronal lineages in the Drosophila central brain
Source: eLife. 2020 Apr 7;9:e53518. doi: 10.7554/eLife.53518 (PMC7173964; doi:10.7554/eLife.53518)

Figure 1-source data 1A-ALv1

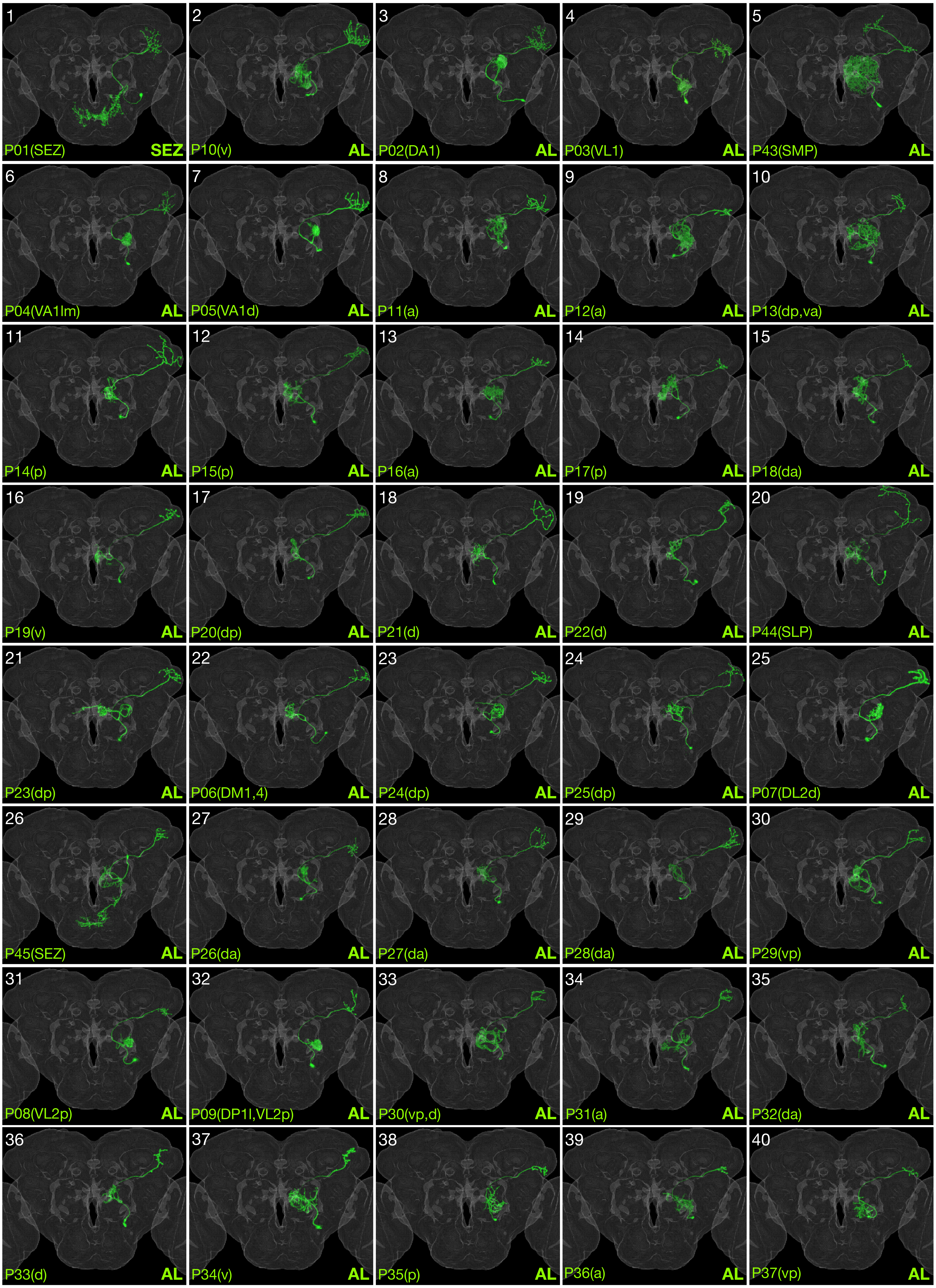

Figure 1-source data 1A-ALv1-cont.

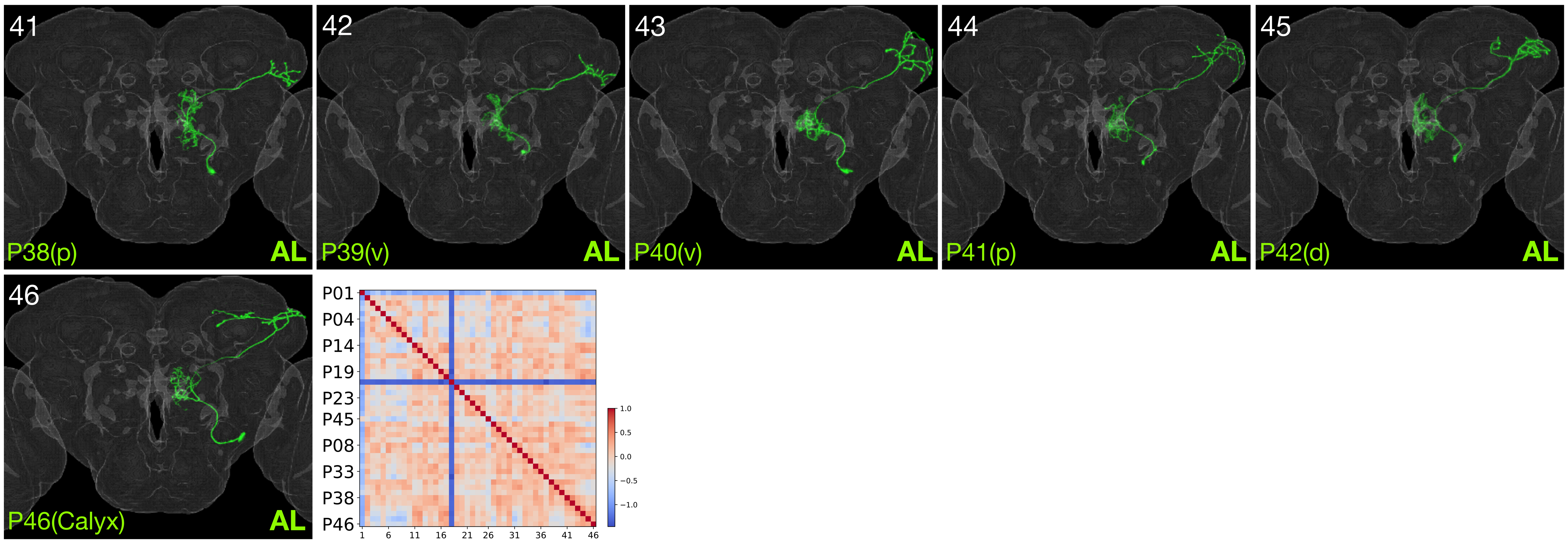

Figure 1-source data 1A-ALv1-cont.

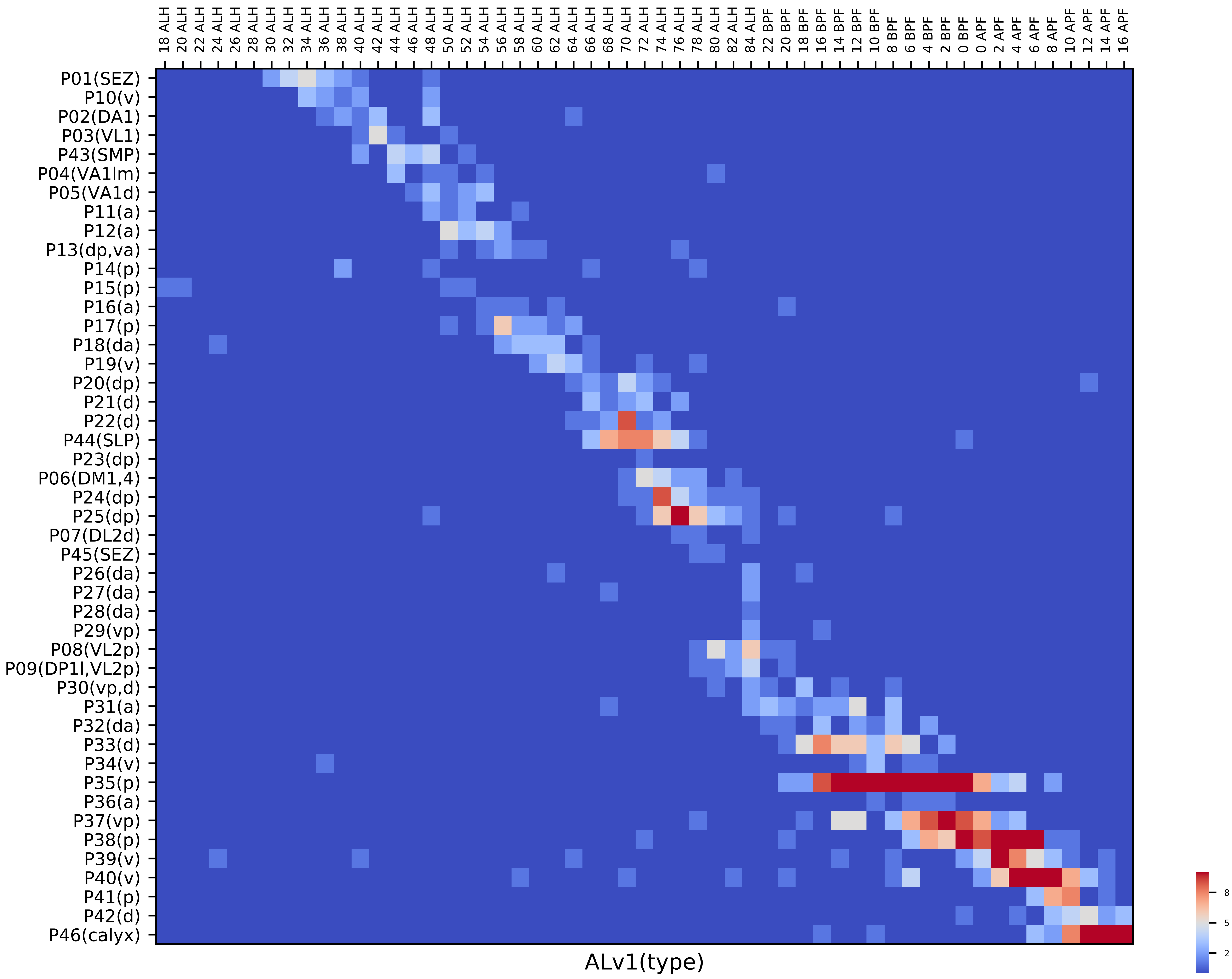

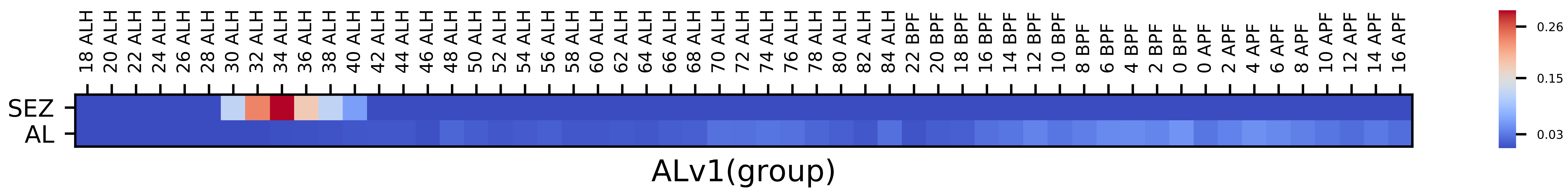

Figure 1-source data 1B-AOTUv1

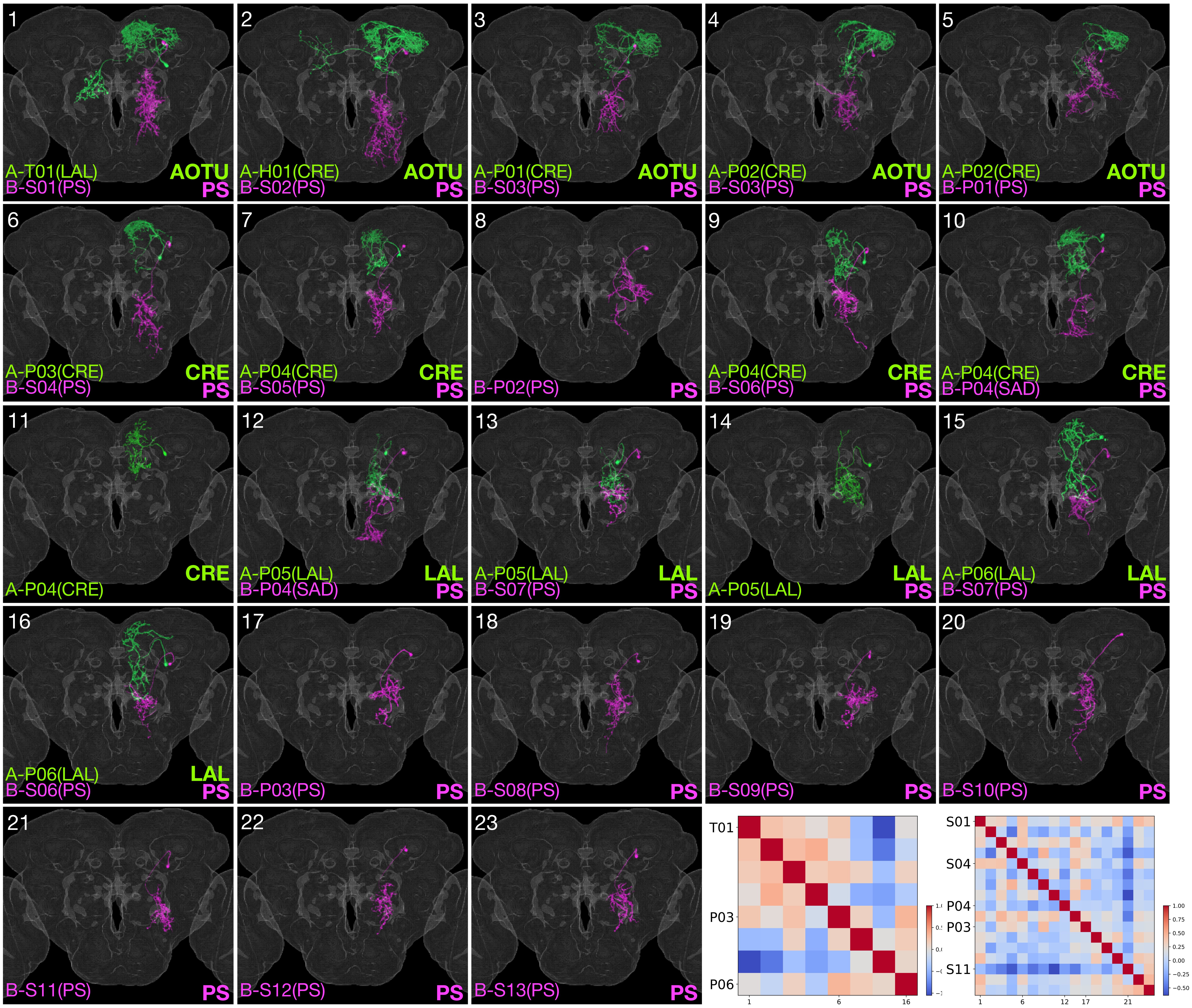

Figure 1-source data 1B-AOTUv1-cont.

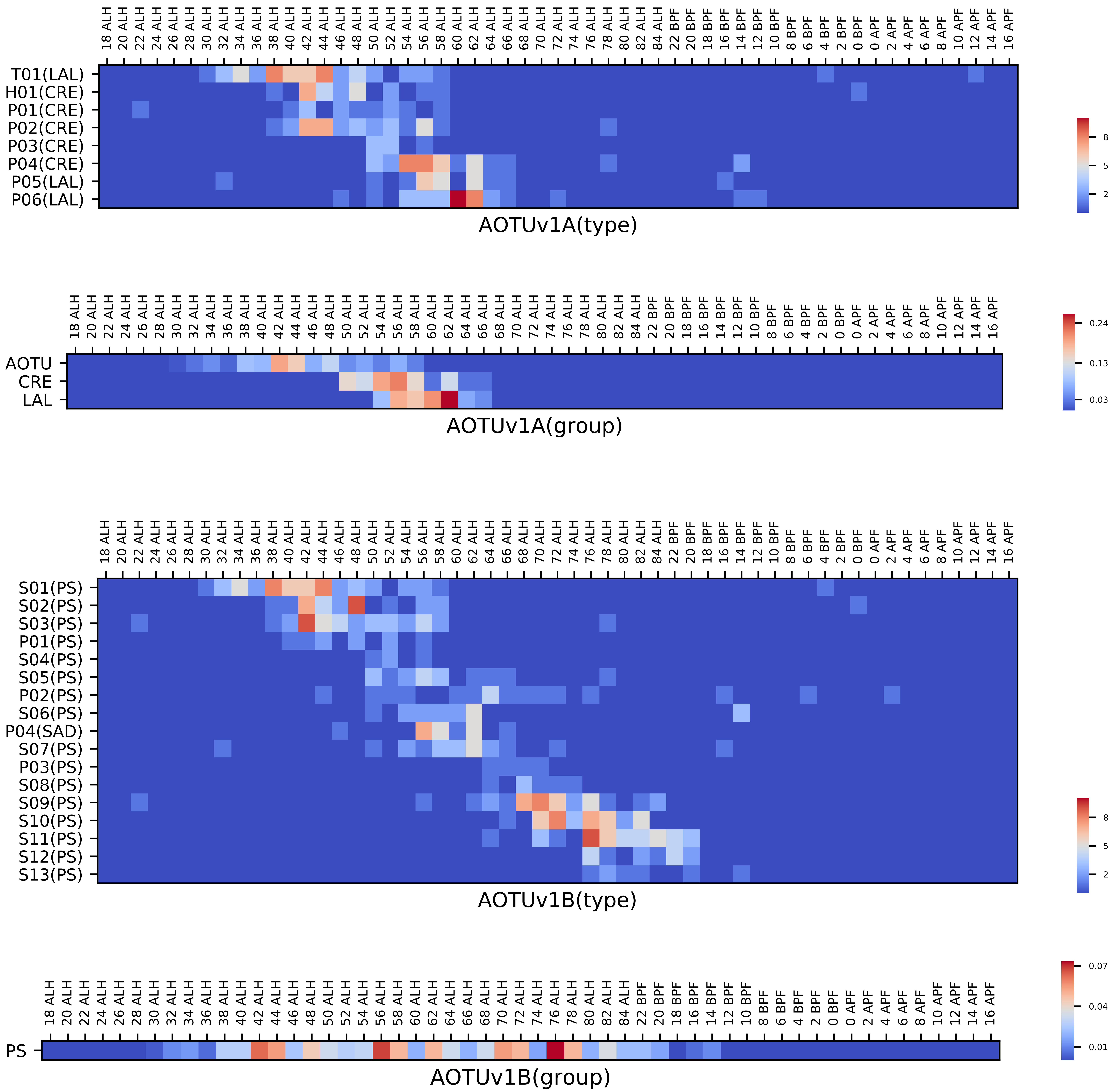

Figure 1-source data 1C-AOTUv3

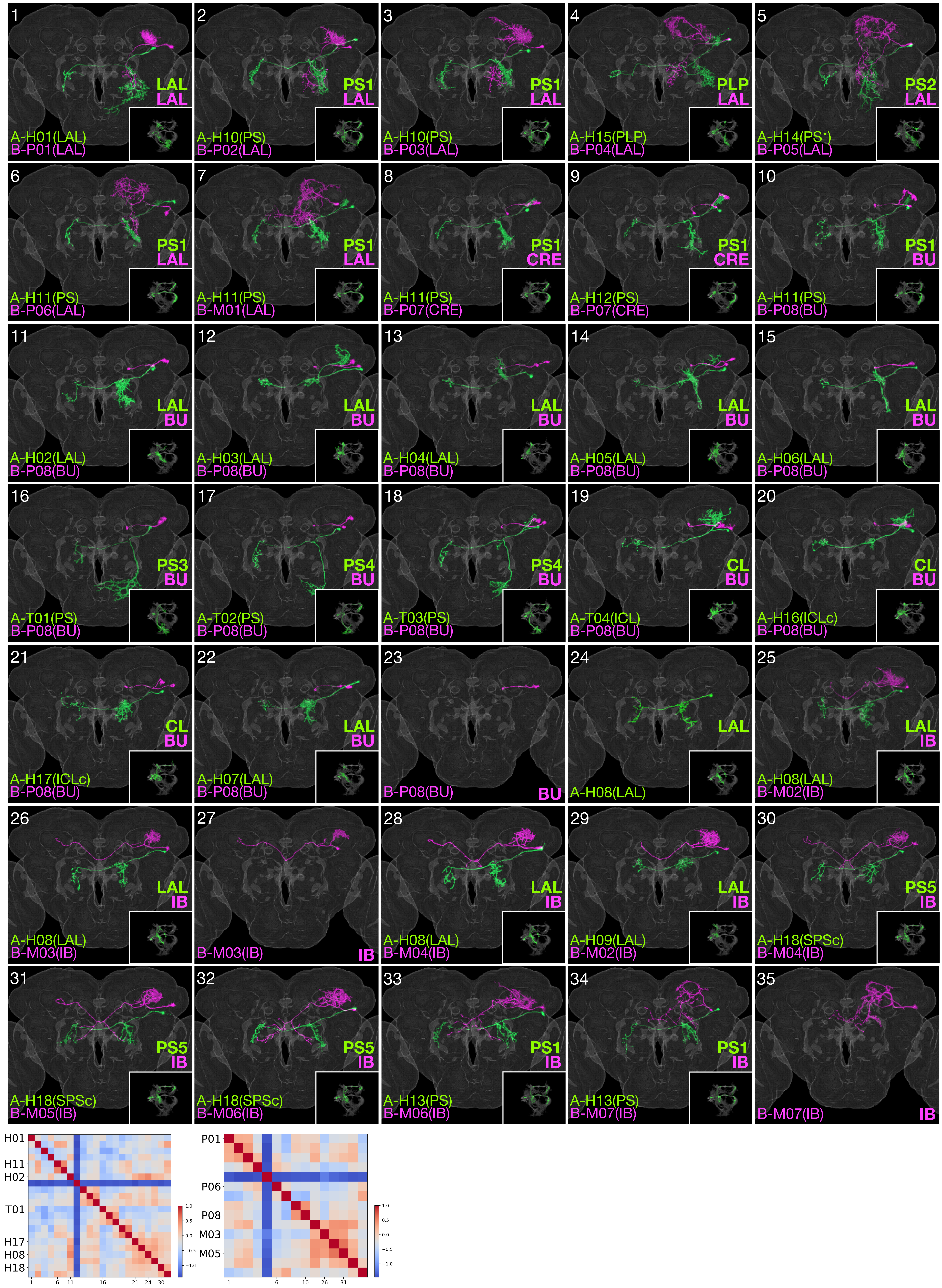

Figure 1-source data 1C-AOTUv3-cont.

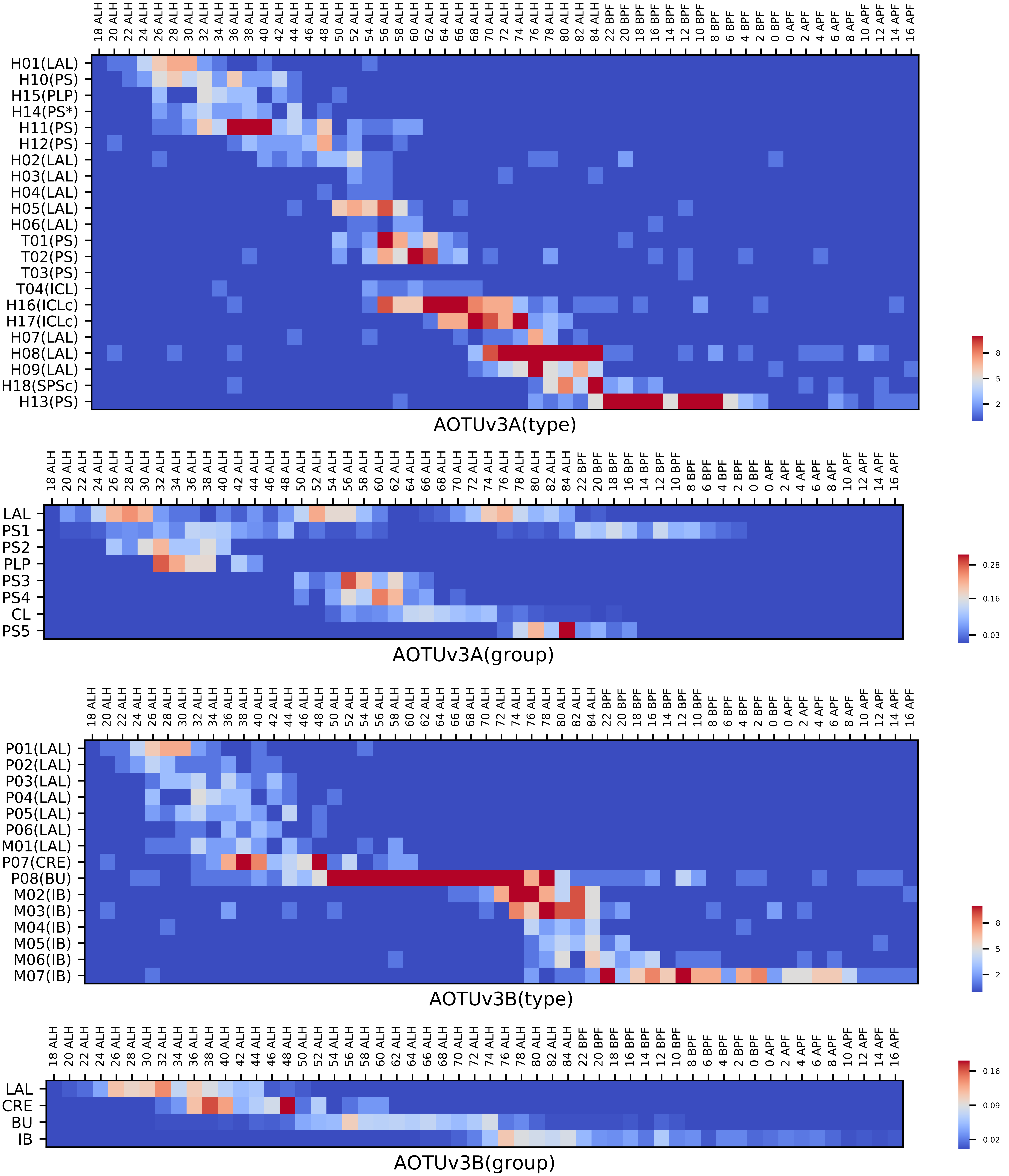

Figure 1-source data 1D-AOTUv4

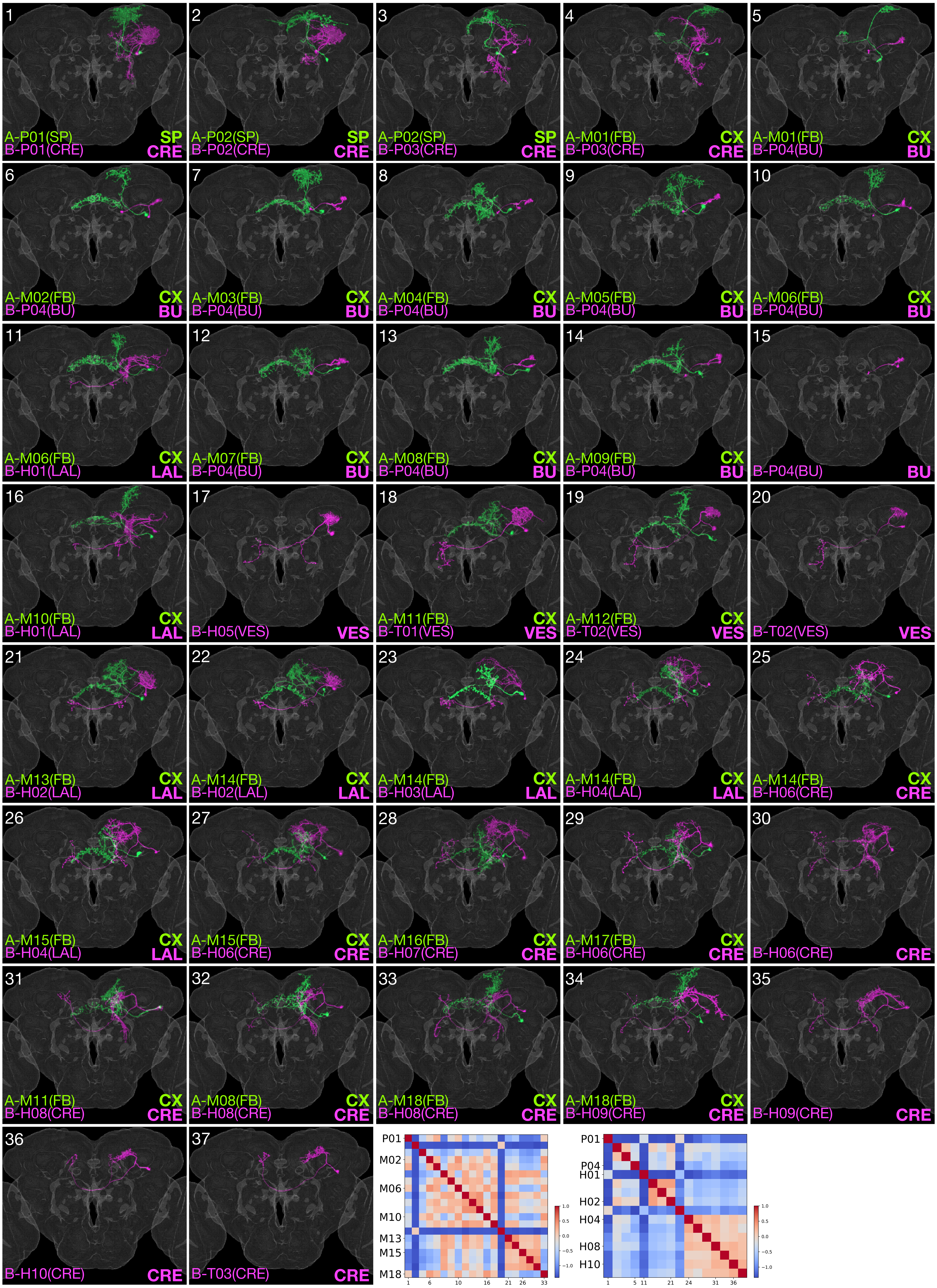

Figure 1-source data 1D-AOTUv4-cont.

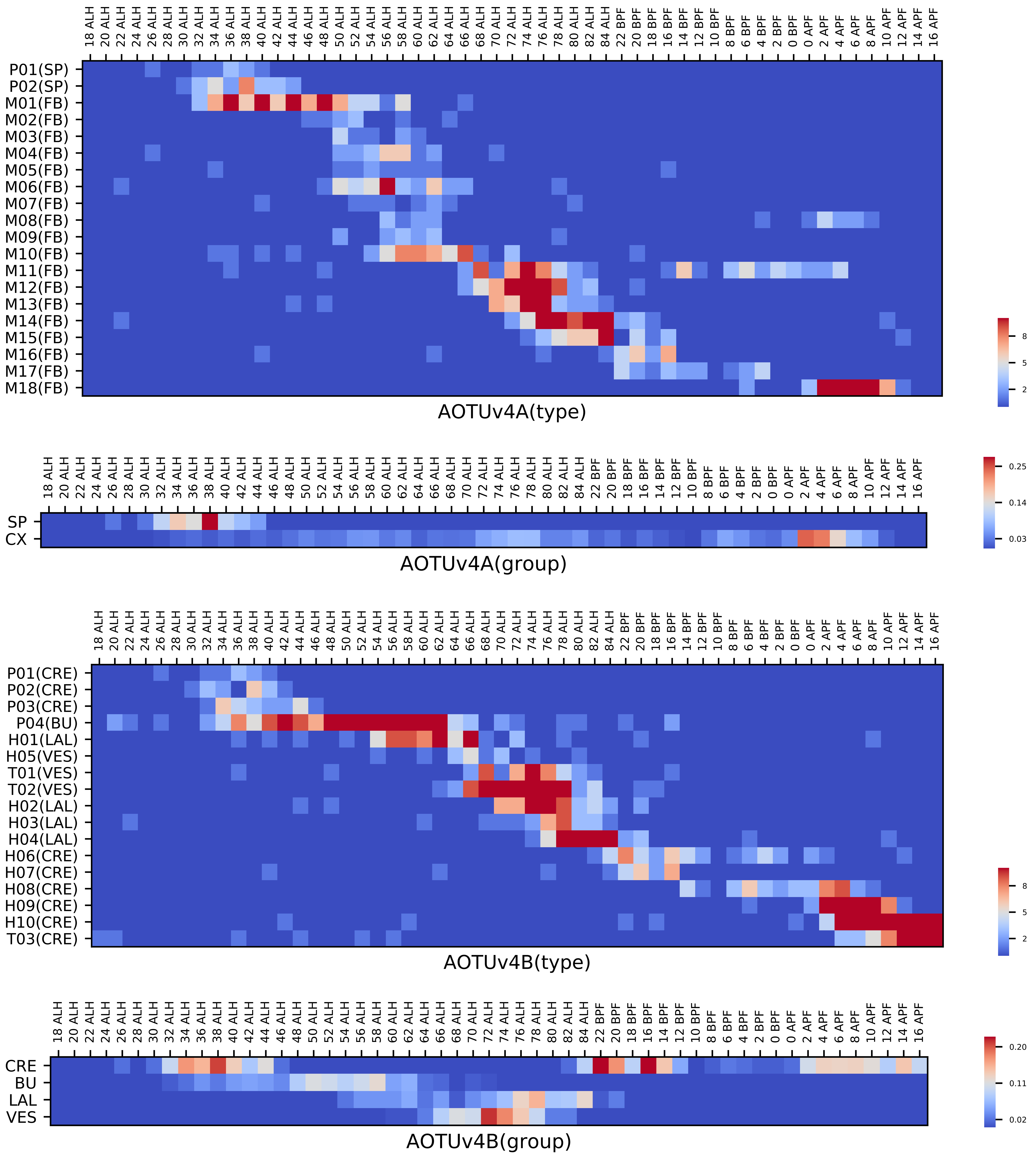

Figure 1-source data 1E-CREa1

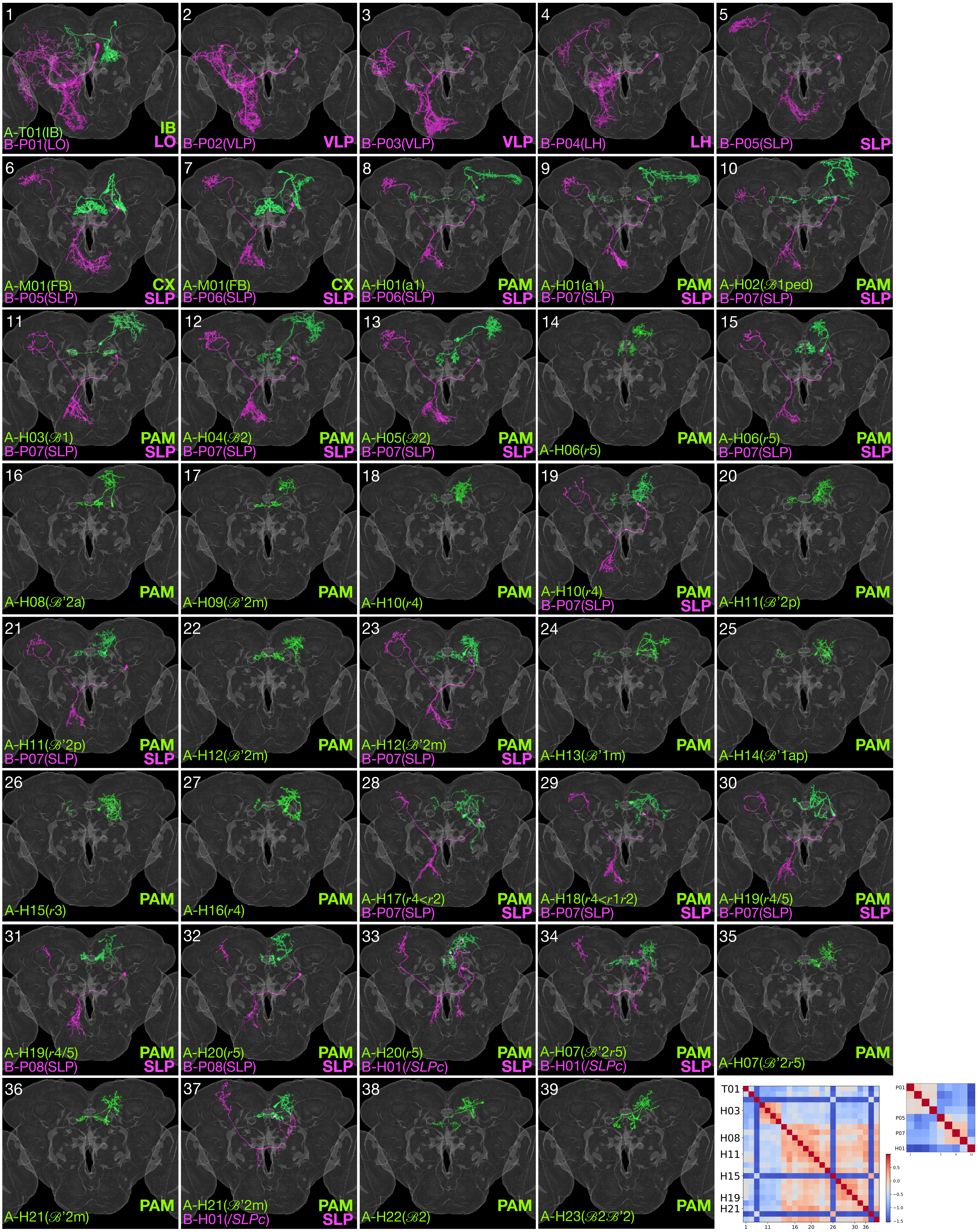

Figure 1-source data 1E-CREa1-cont.

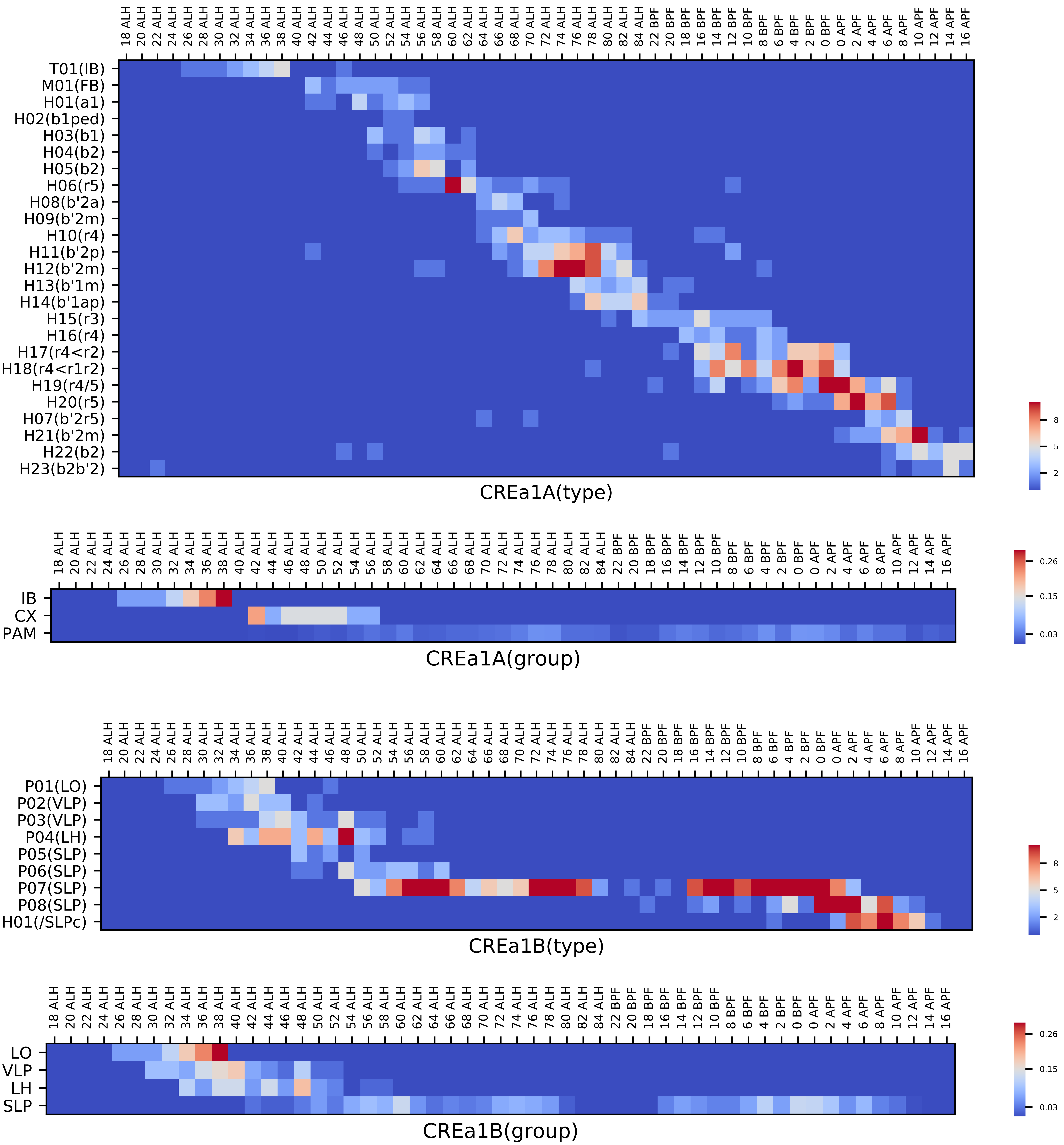

Figure 1-source data 1F-CREa2

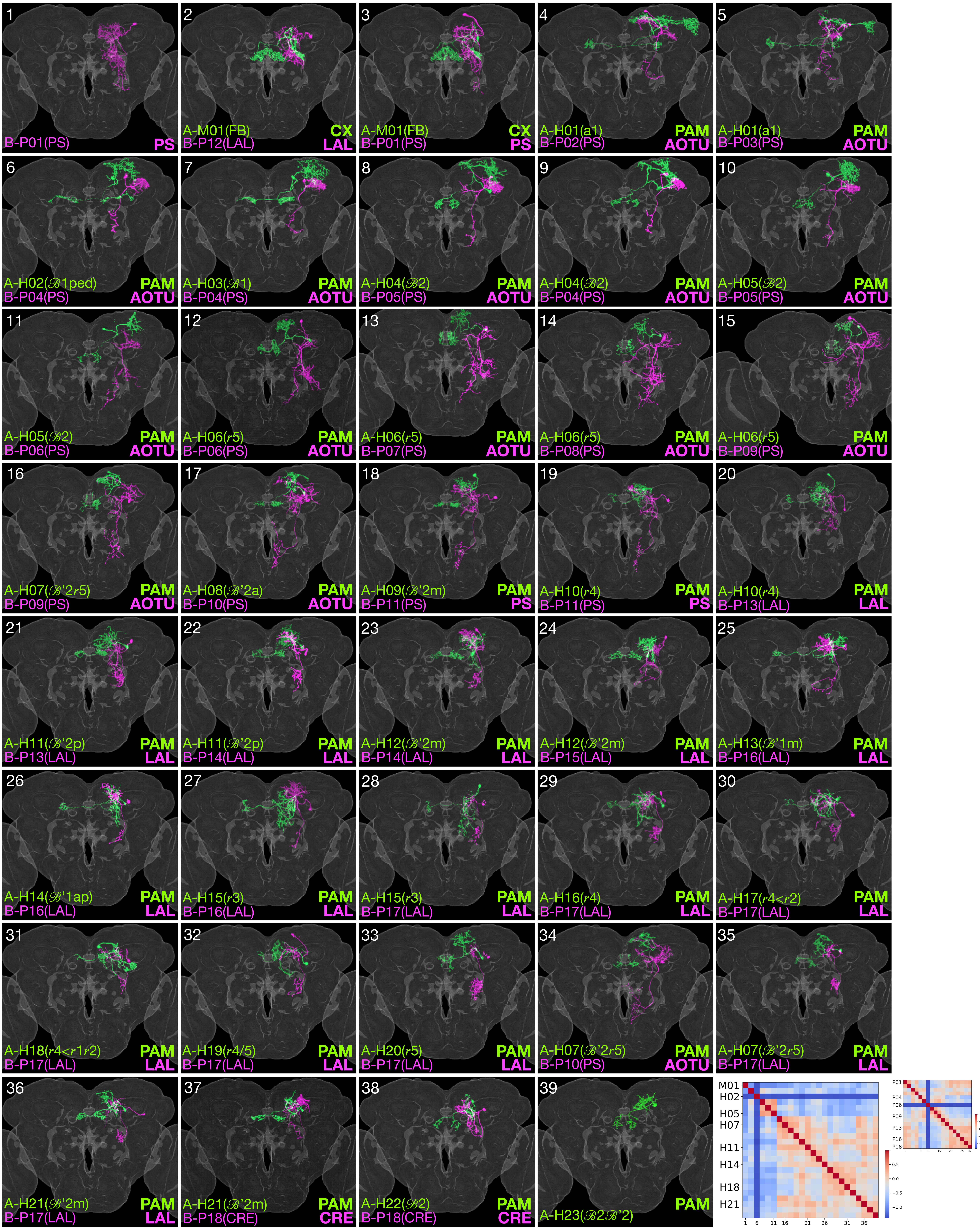

Figure 1-source data 1F-CREa2-cont.

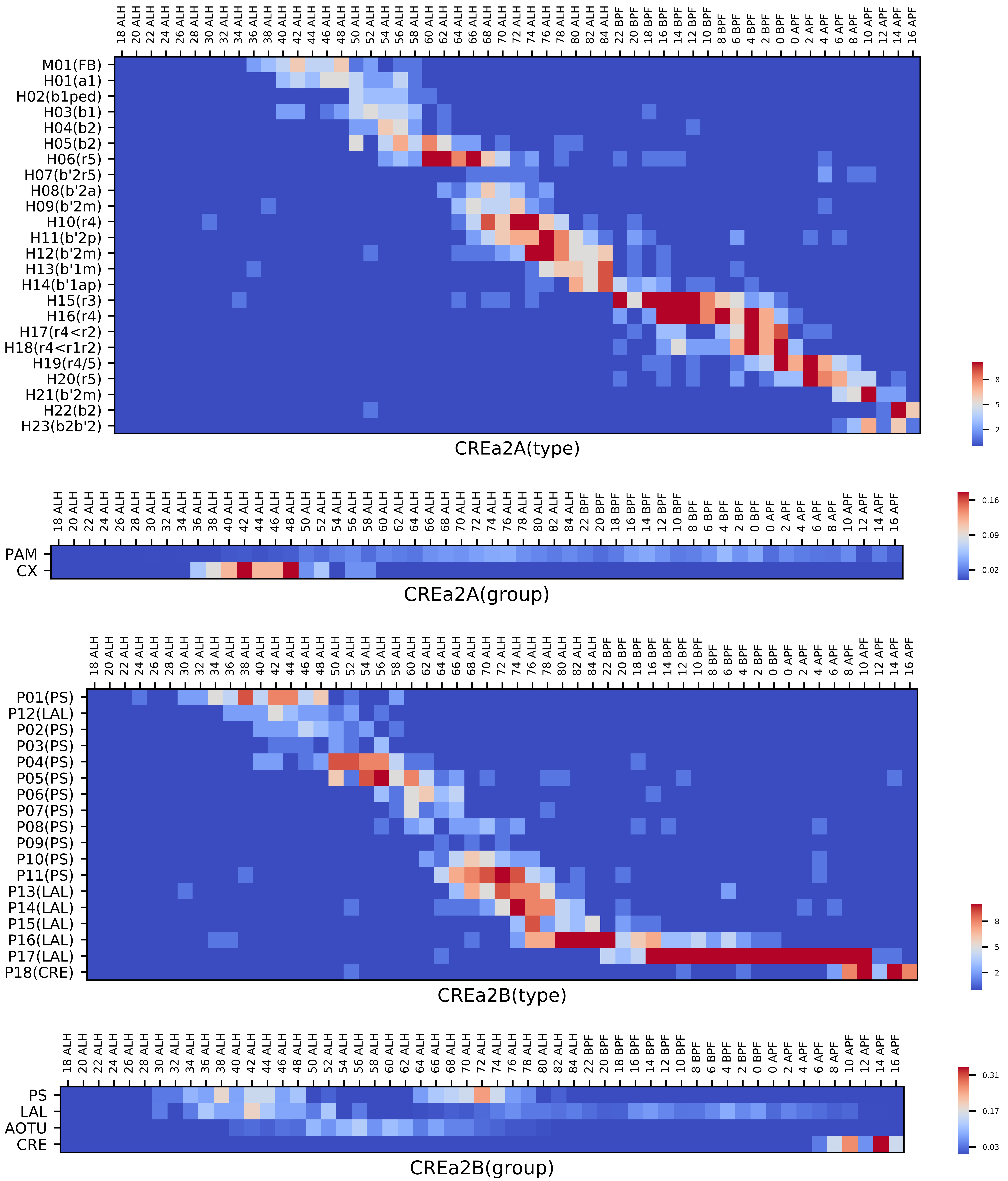

Figure 1-source data 1G-FLAa1

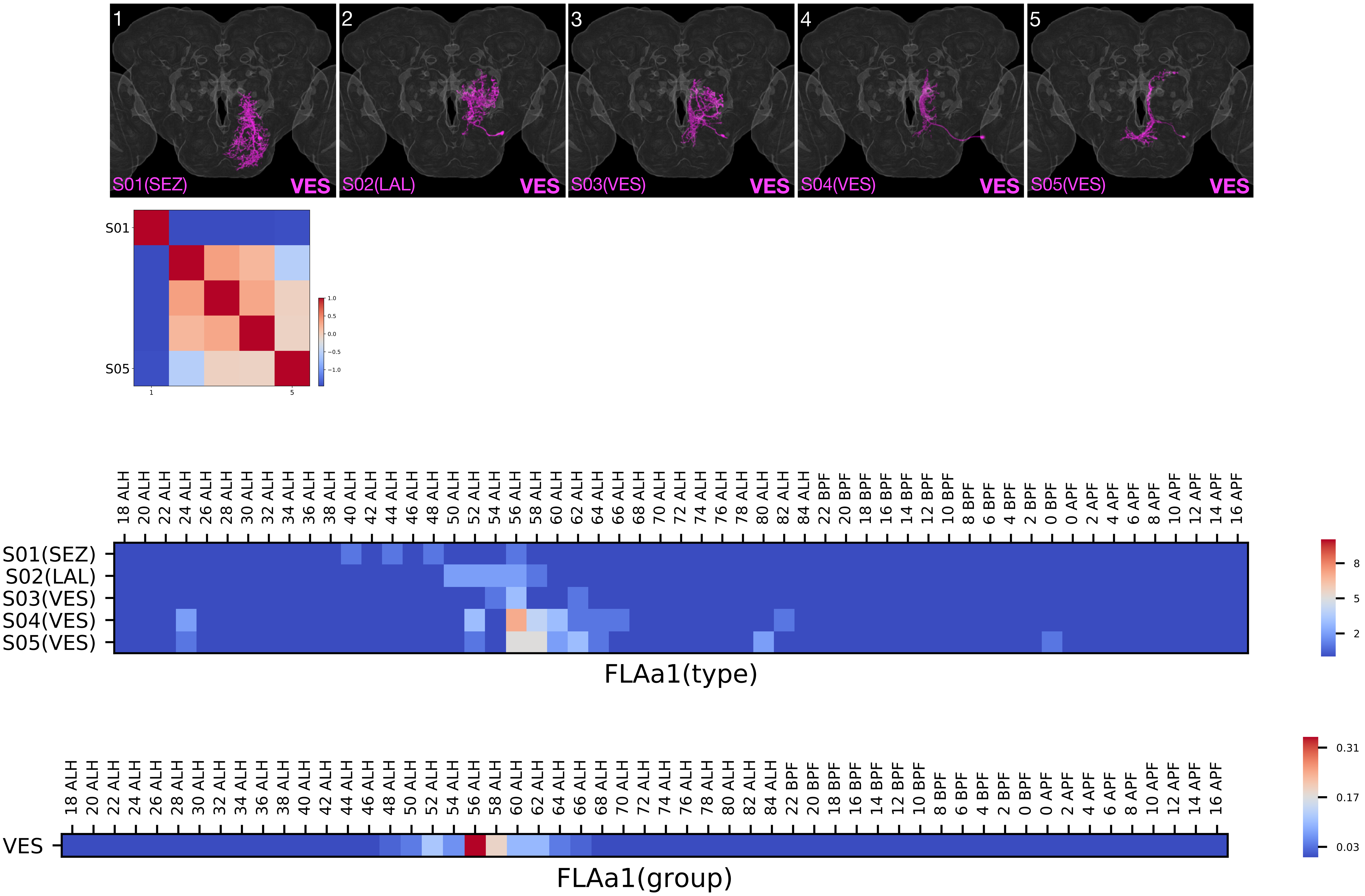

Figure 1-source data 1H-FLAa2

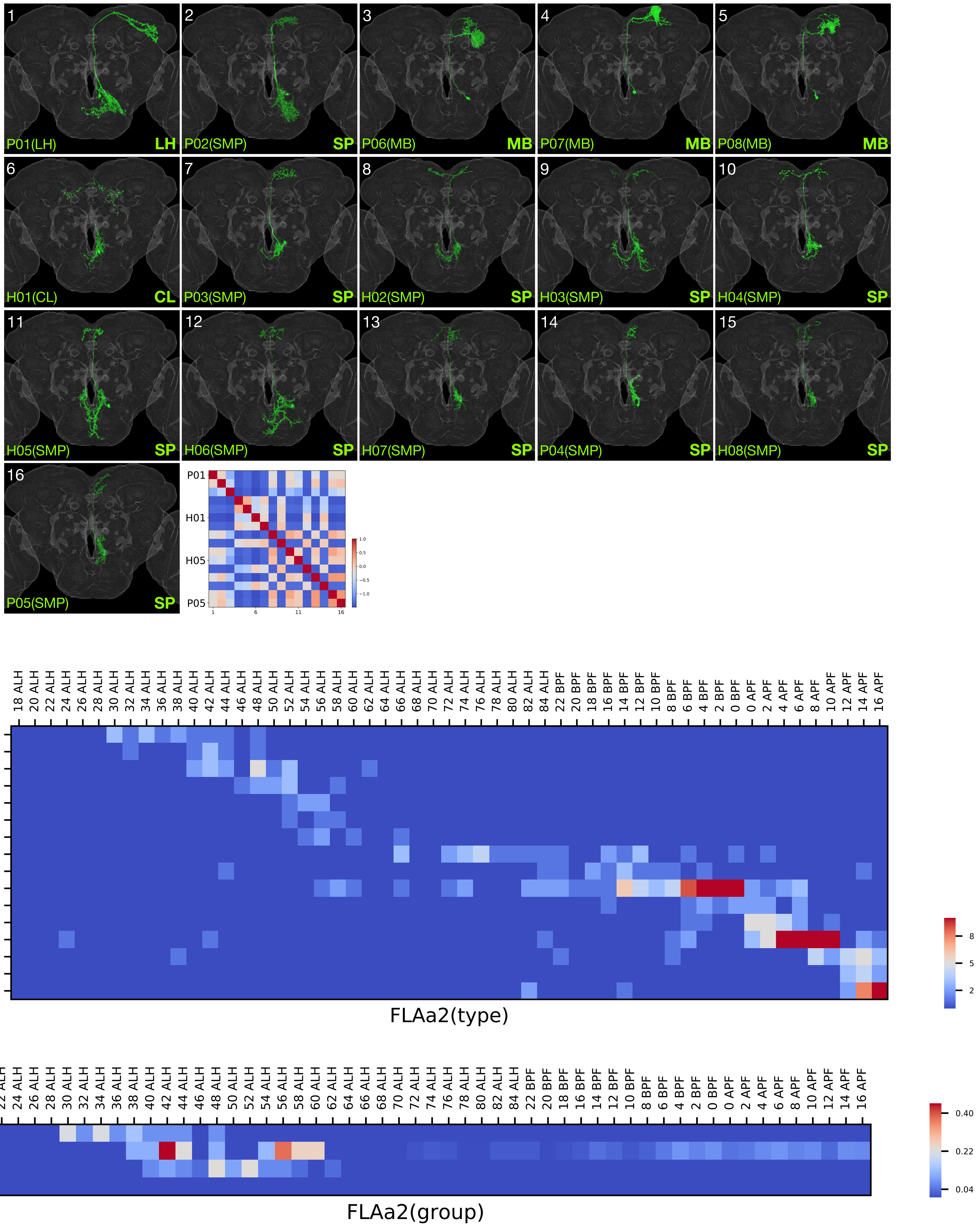

Figure 1-source data 1I-FLAa3

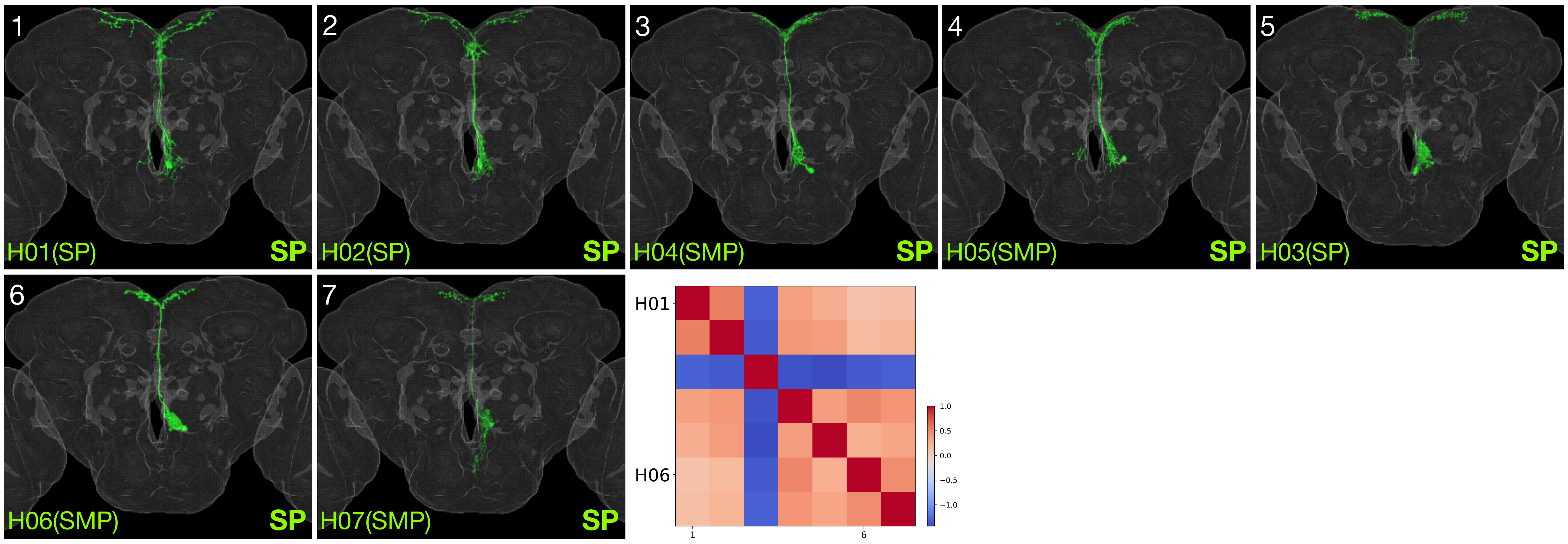

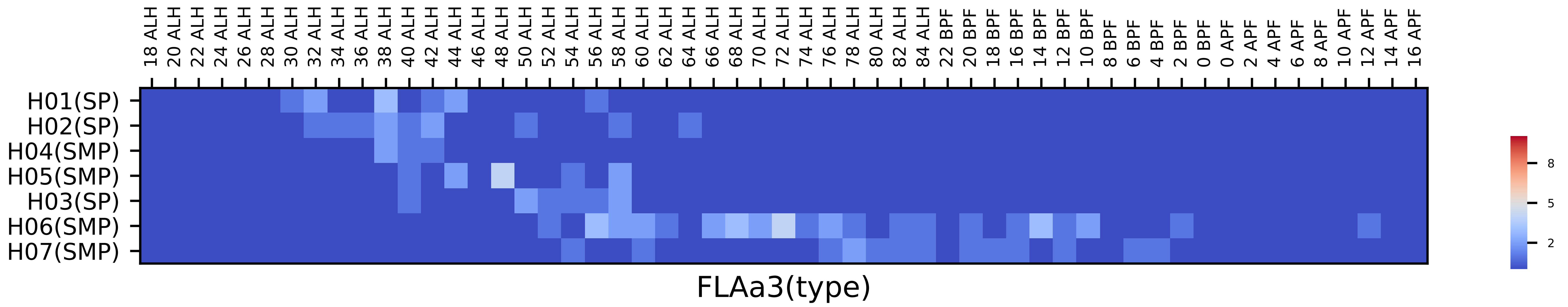

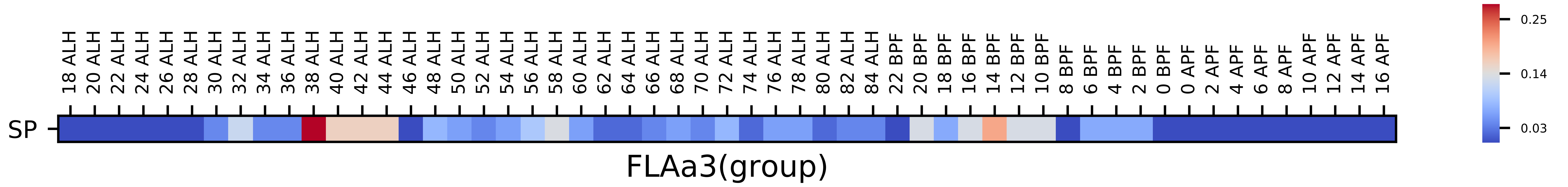

Figure 1-source data 1J-LALv1

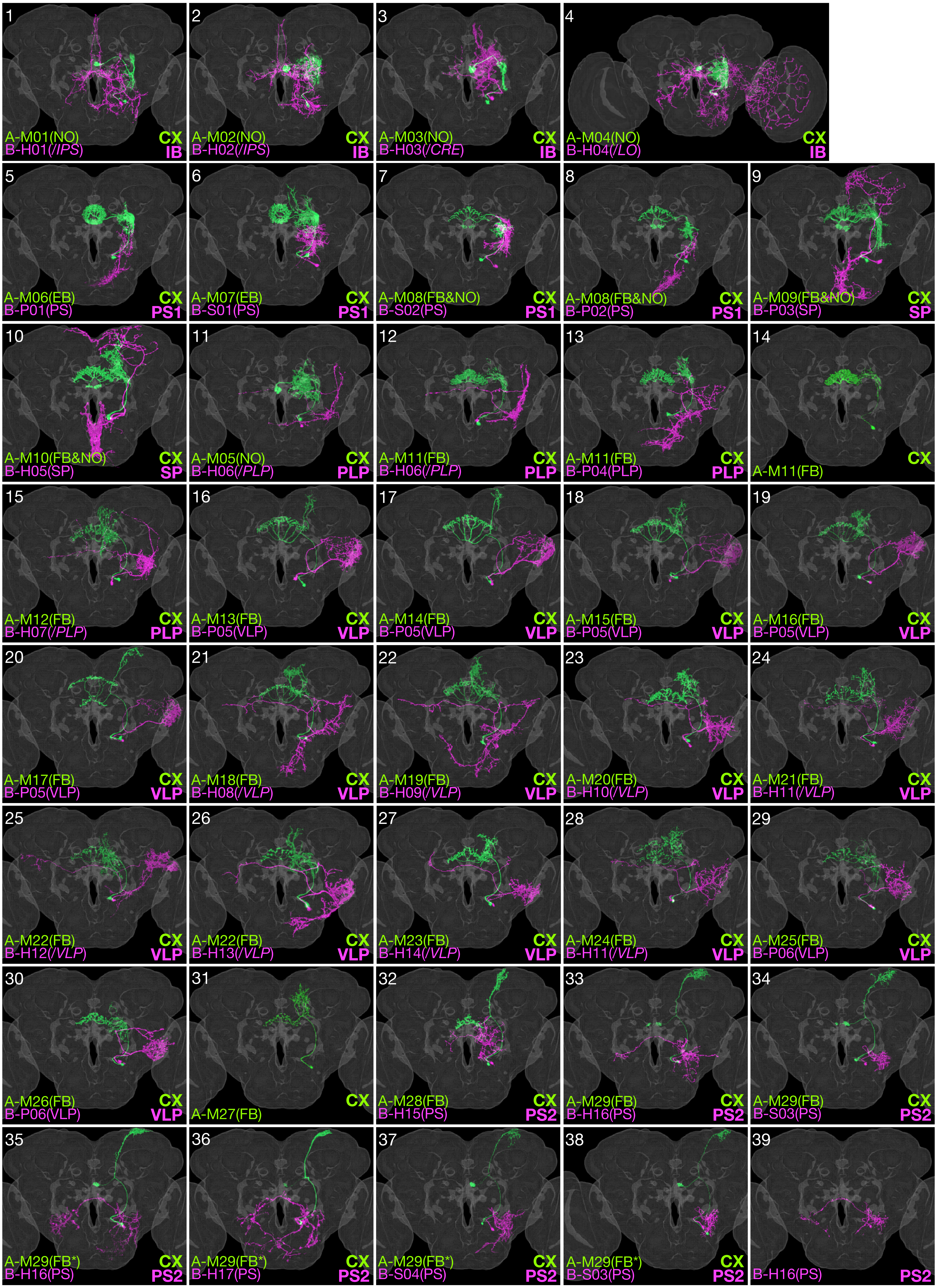

Figure 1-source data 1J-LALv1-cont.

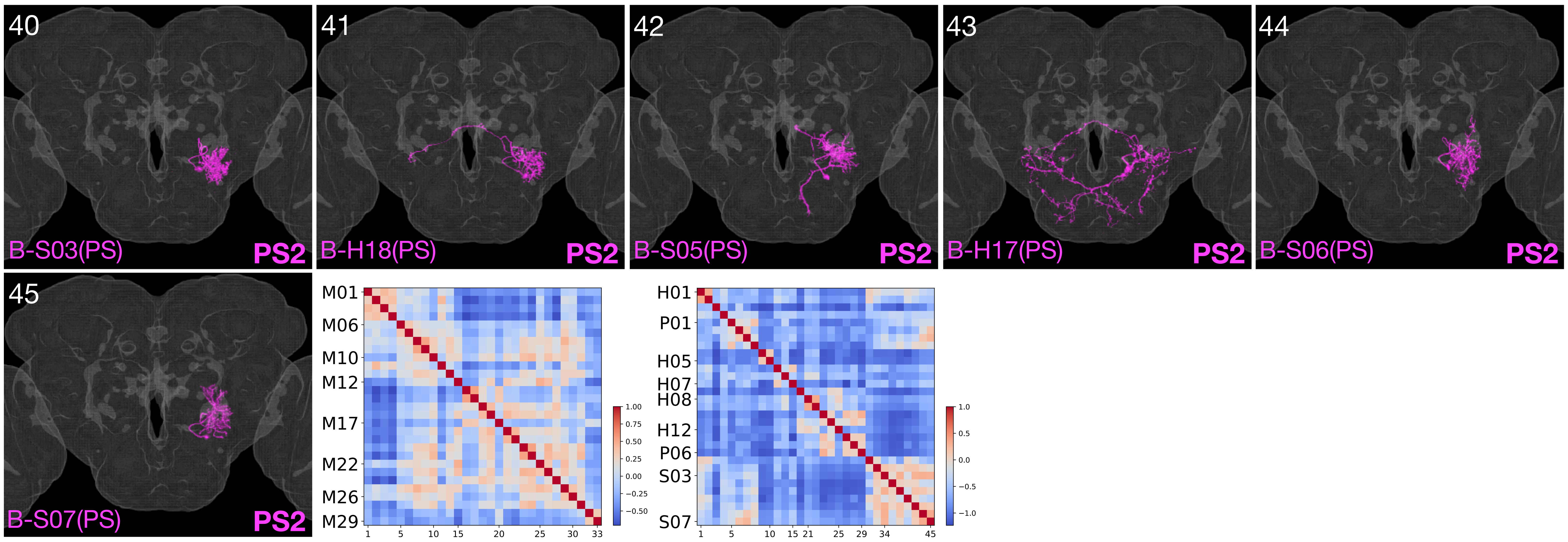

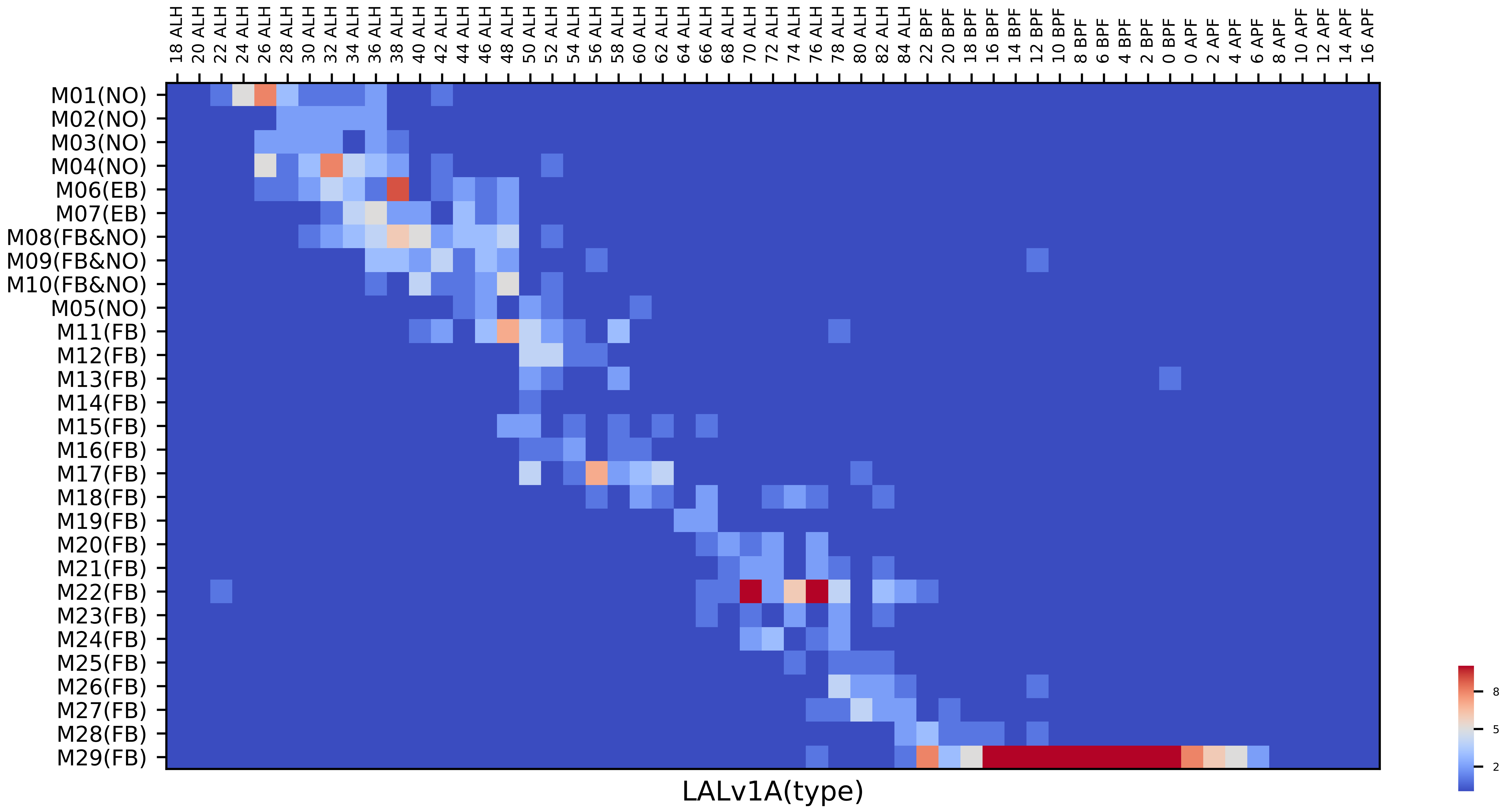

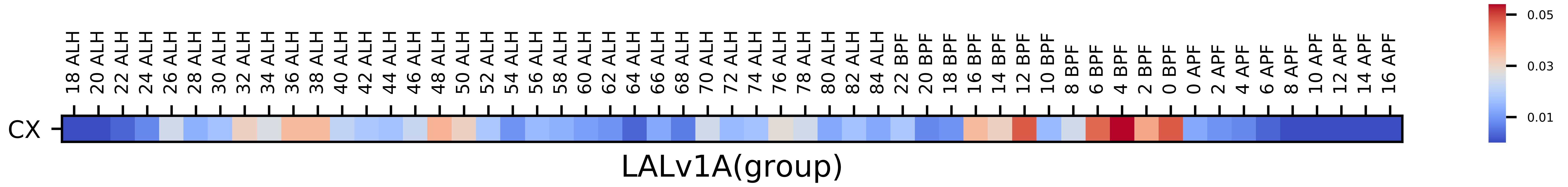

Figure 1-source data 1J-LALv1-cont.

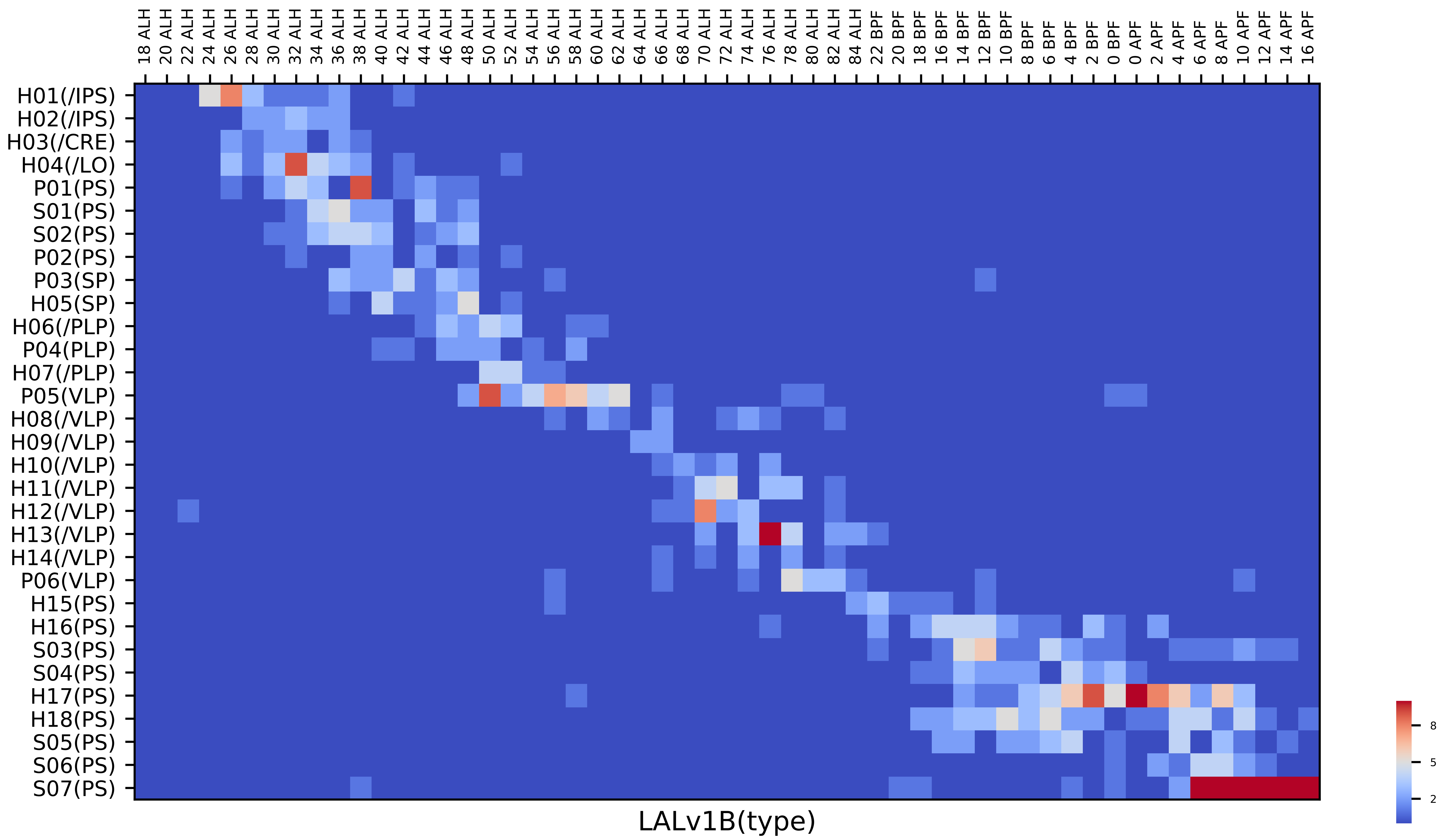

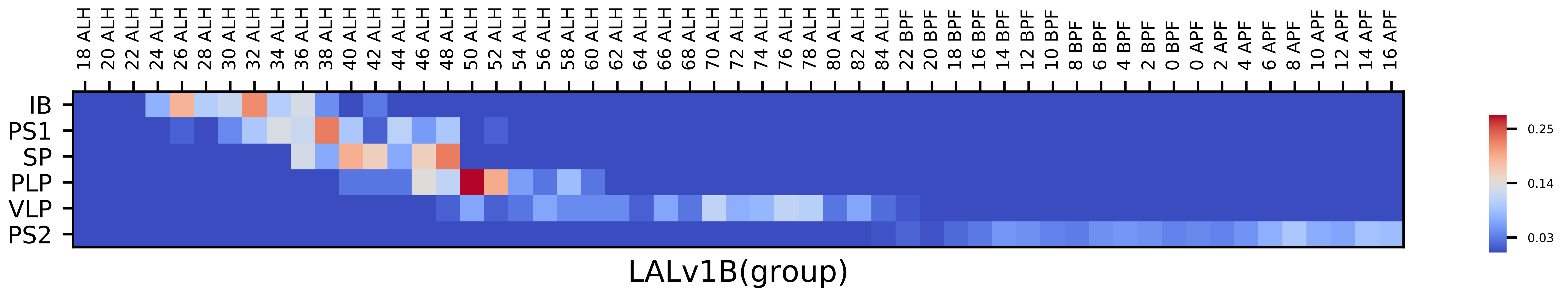

Figure 1-source data 1K-SLPpm3

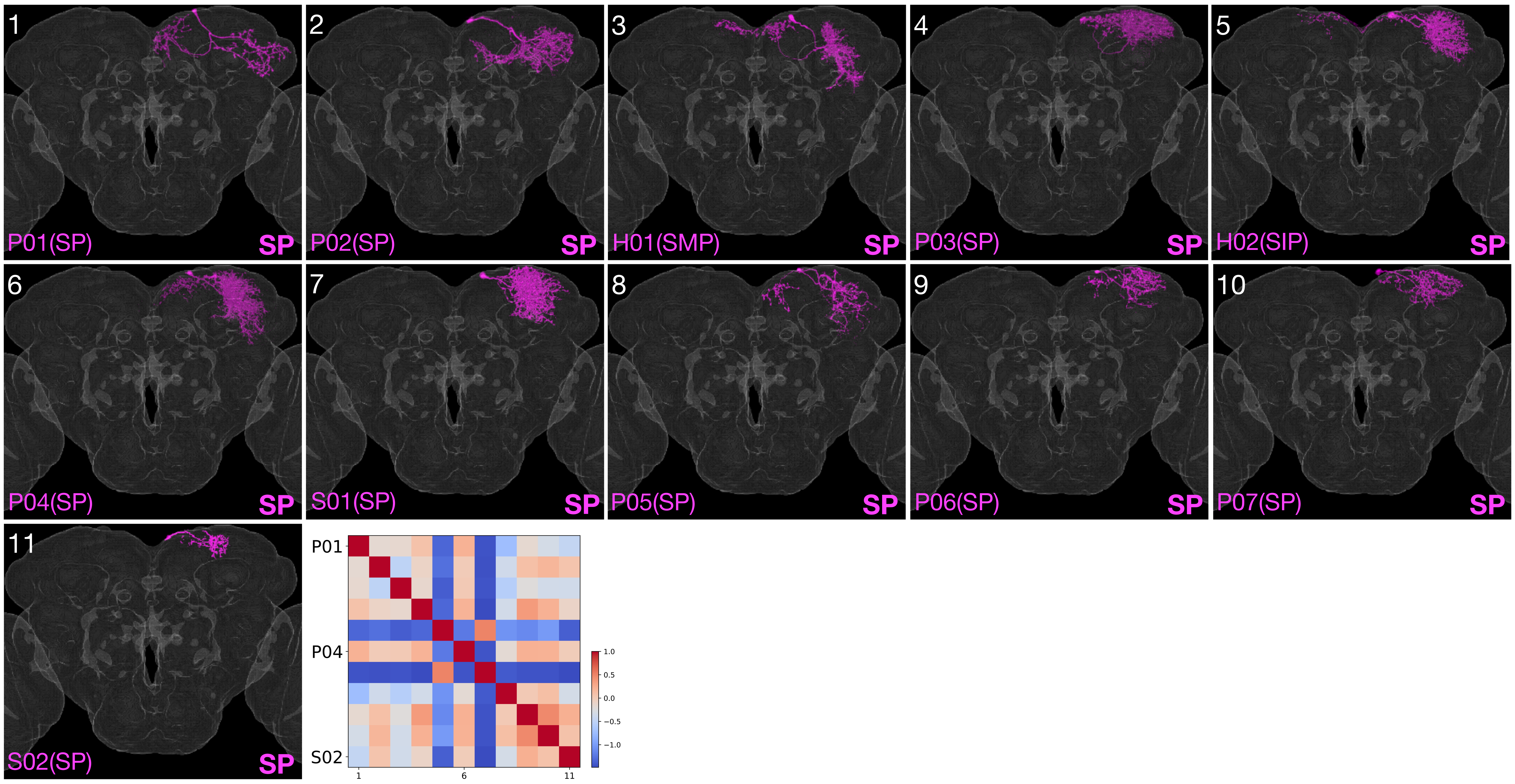

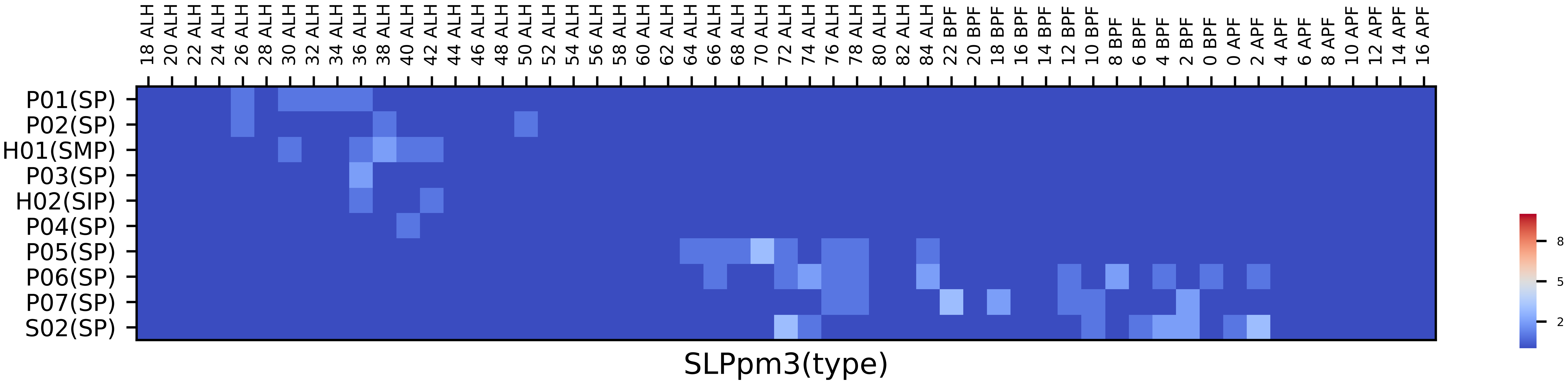

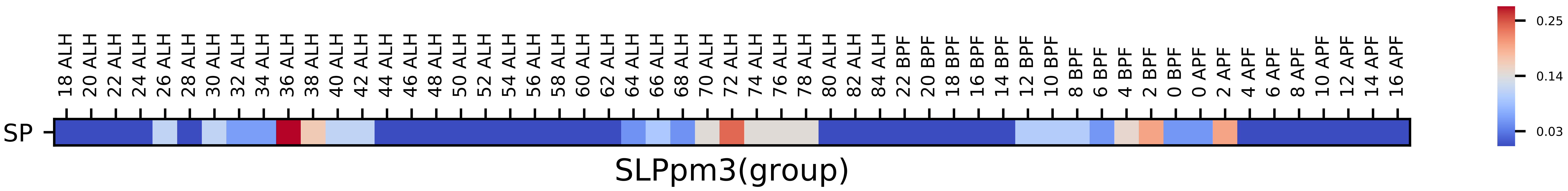

Figure 1-source data 1L-SMPad1

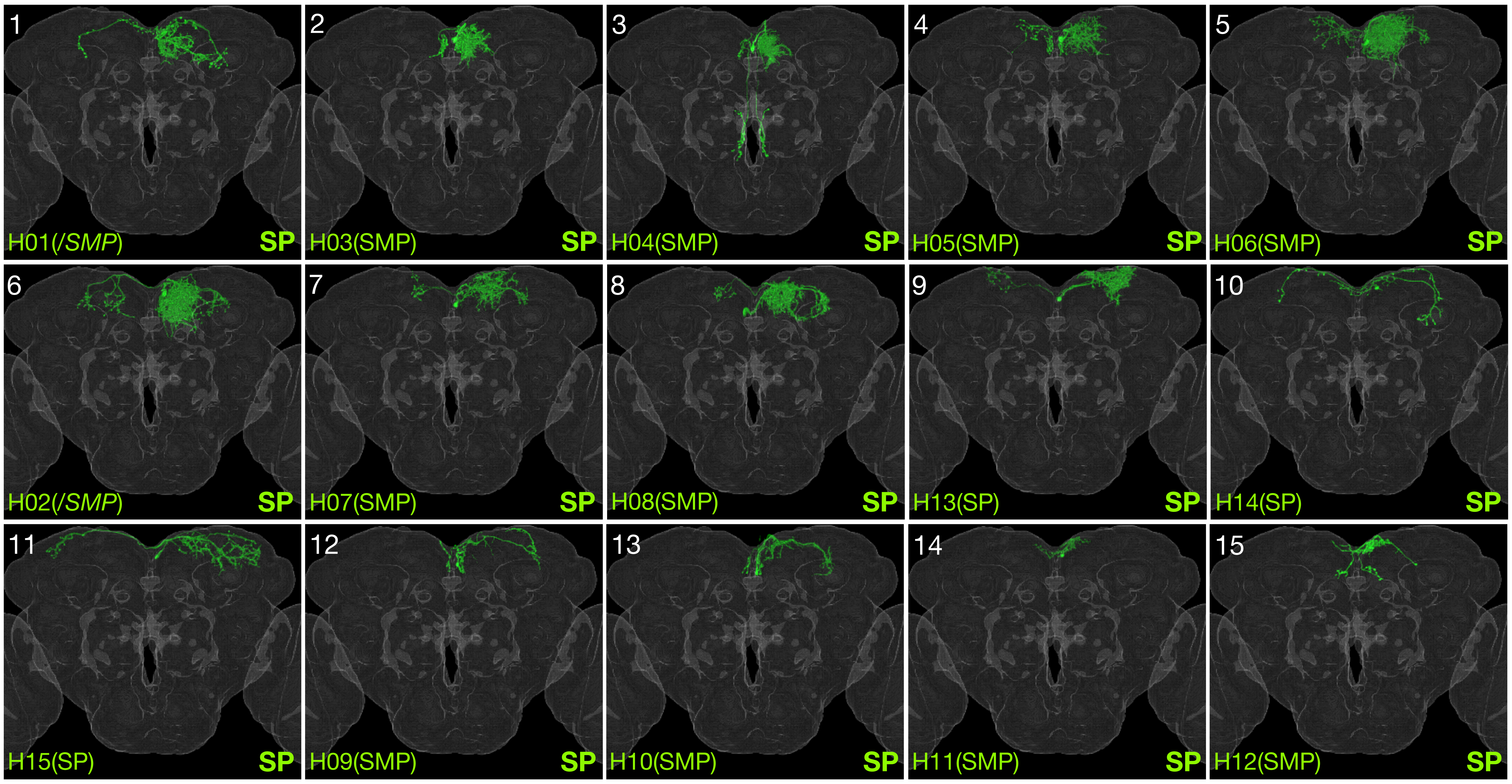

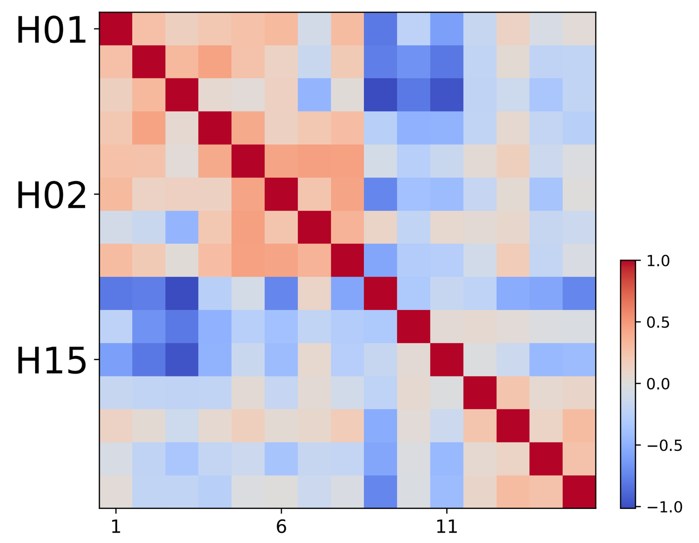

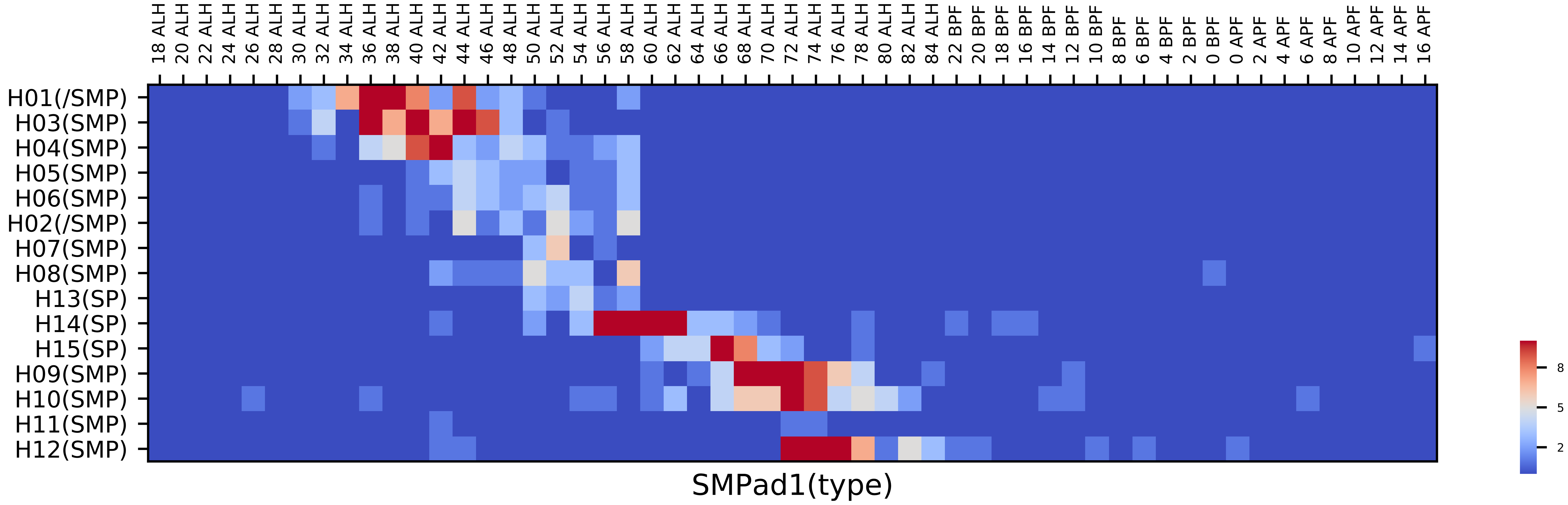

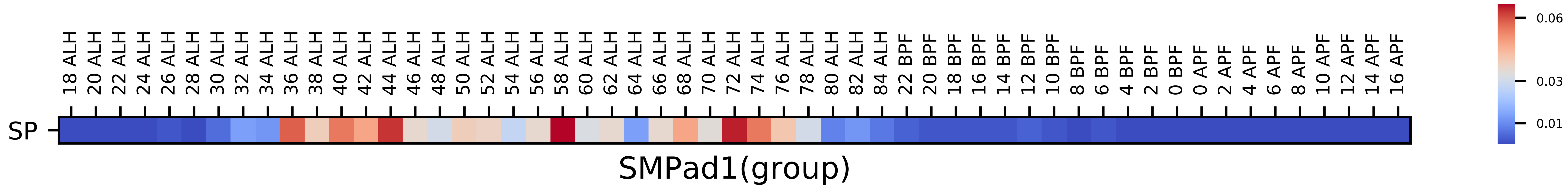

Figure 1-source data 1M-SMPp&v1

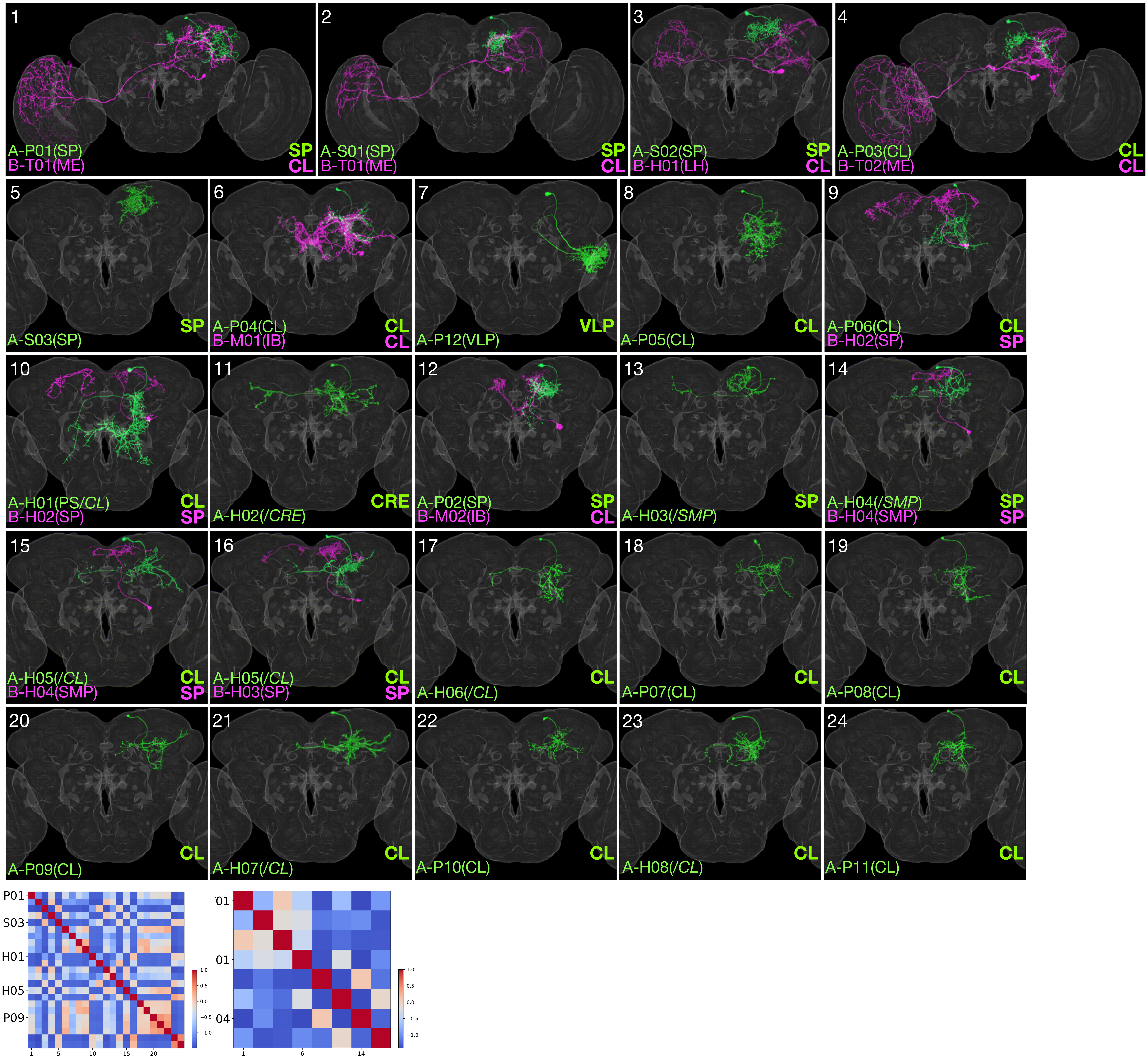

Figure 1-source data 1M-SMPp&v1-cont.

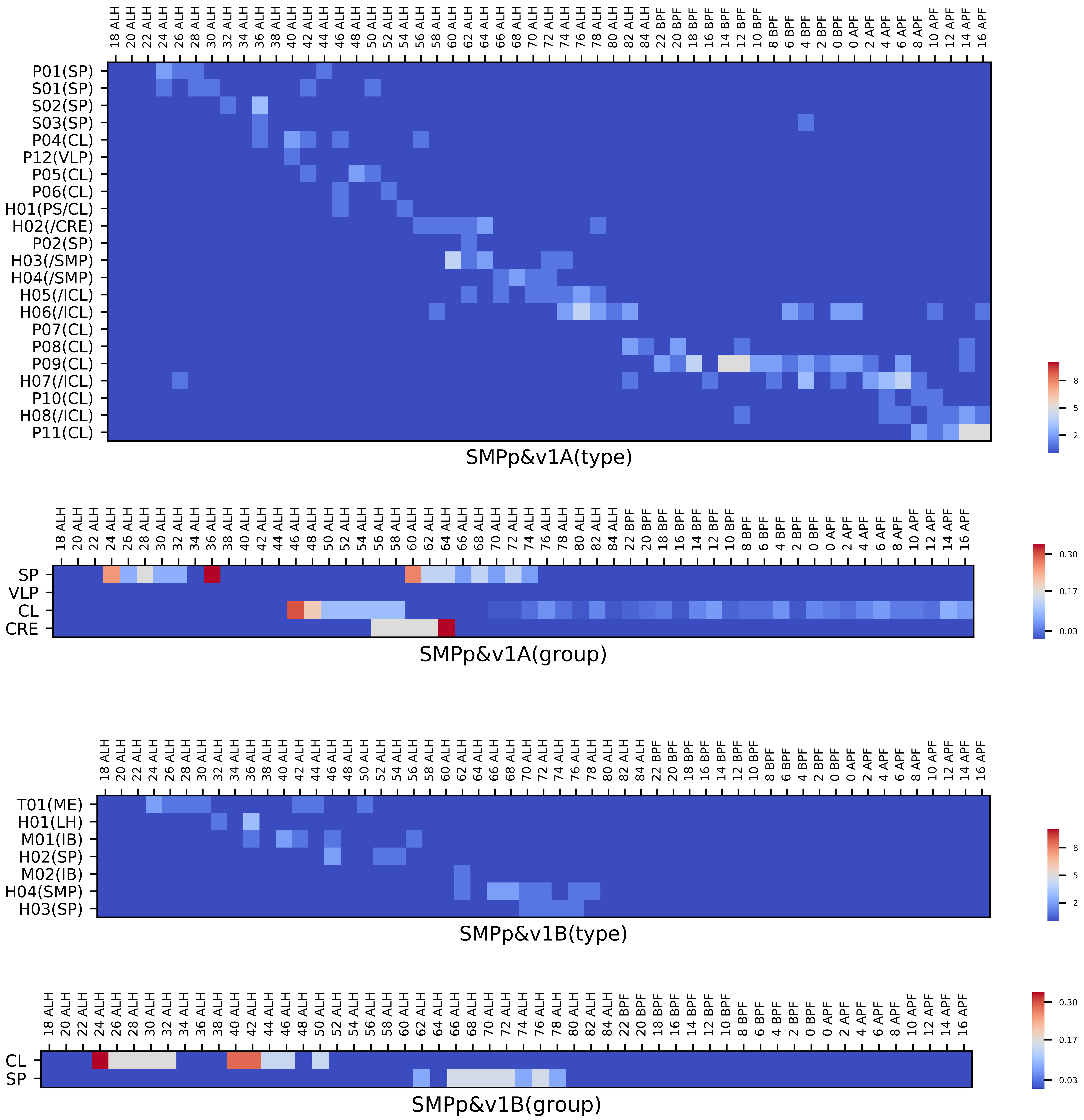

Figure 1-source data 1N-VESa1

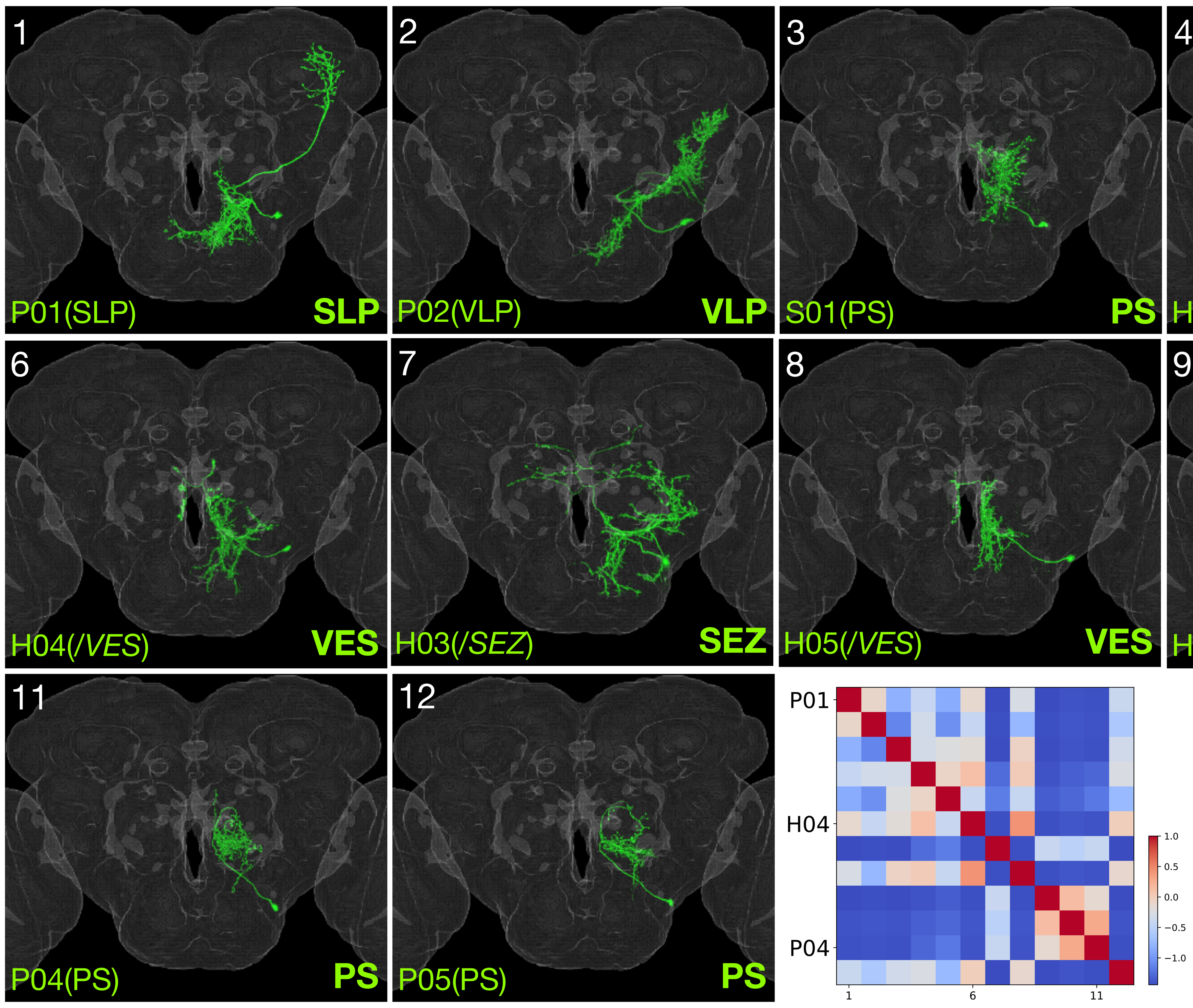

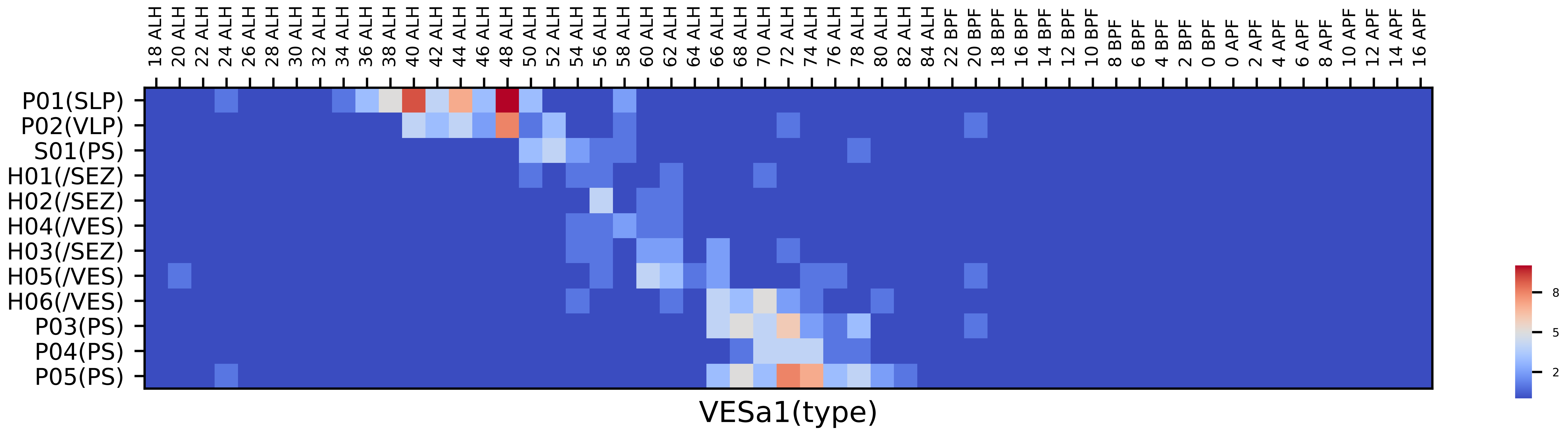

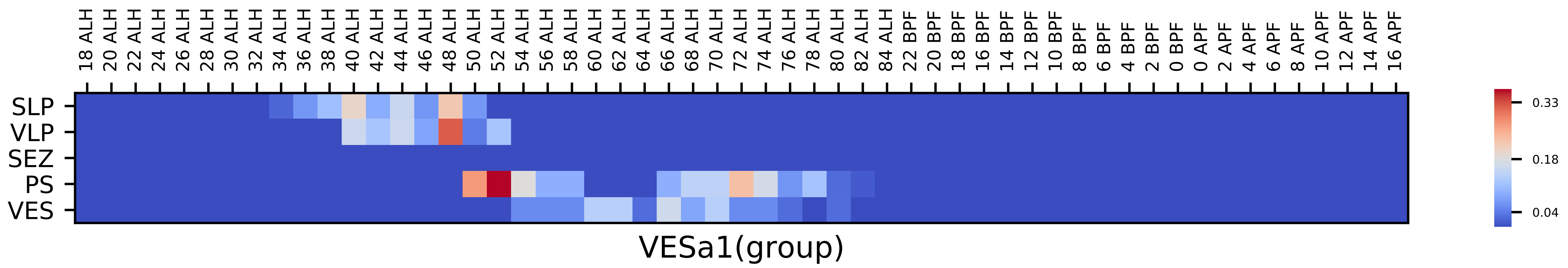

Figure 1-source data 1O-VESa2

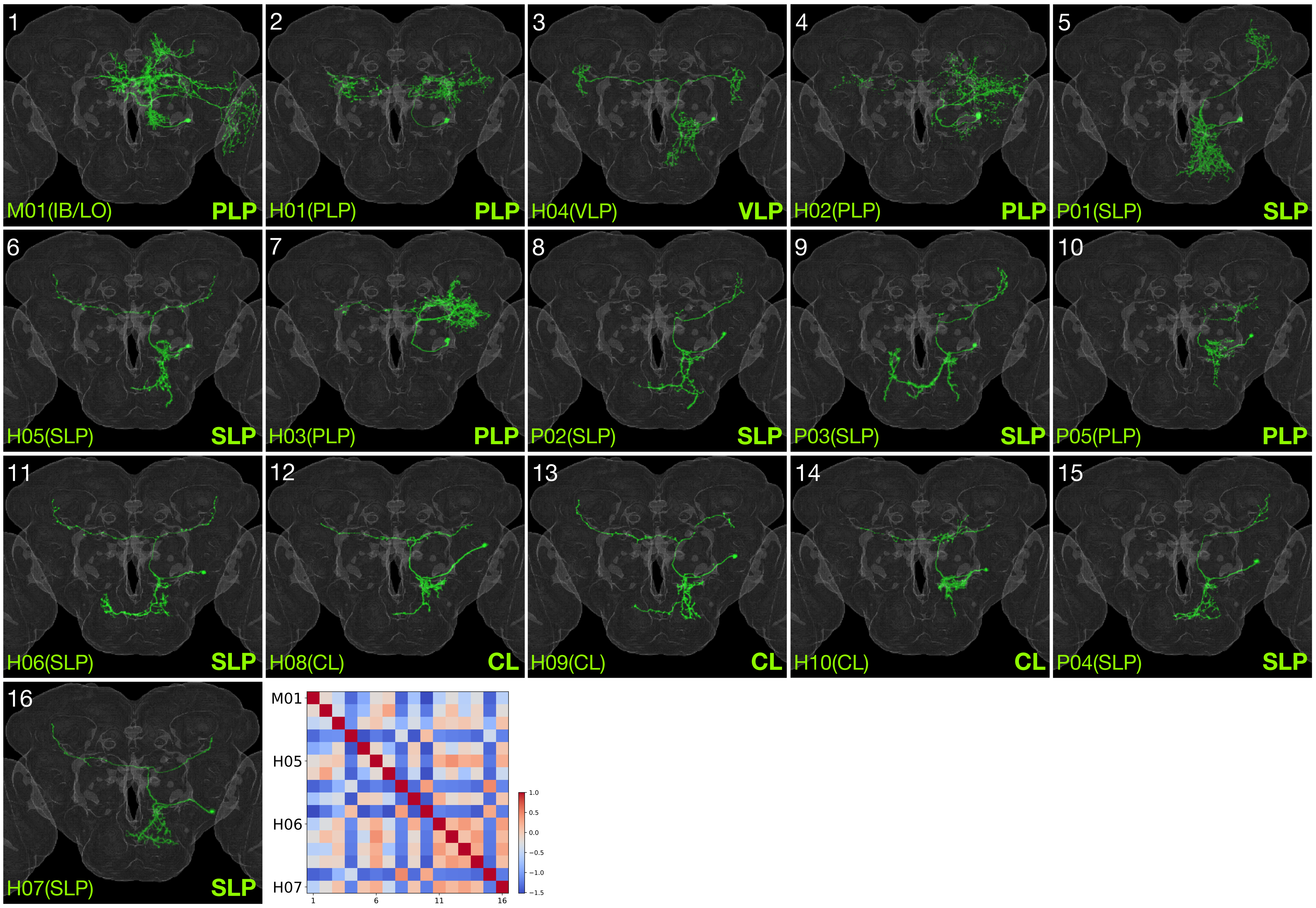

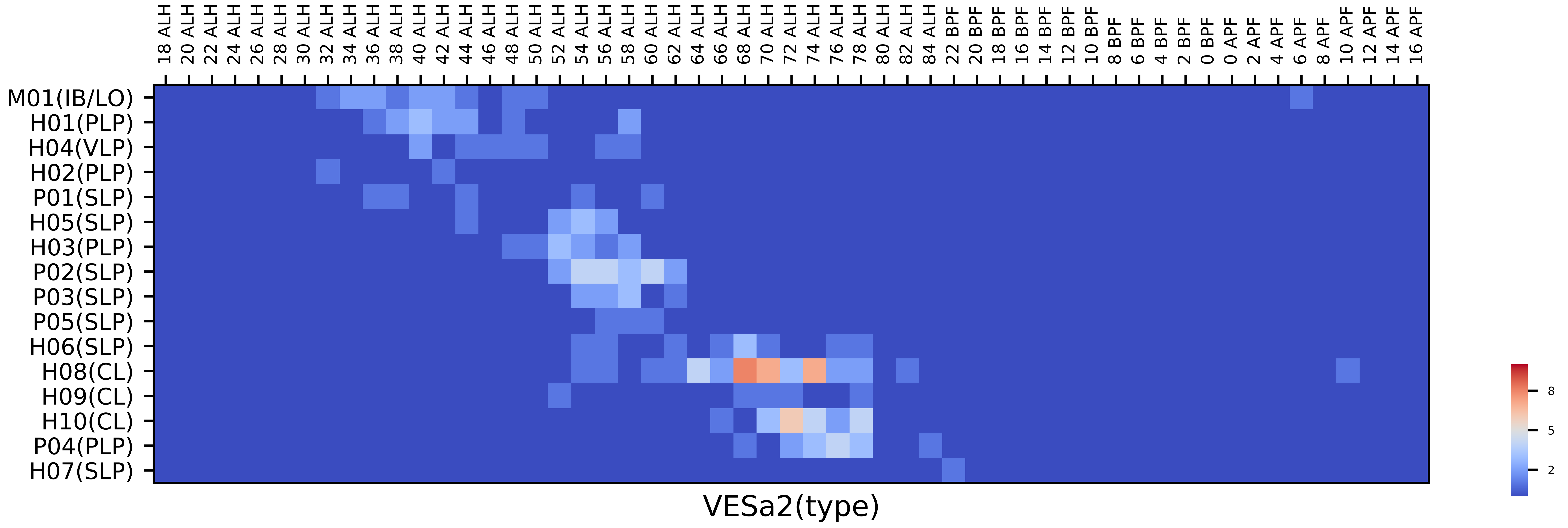

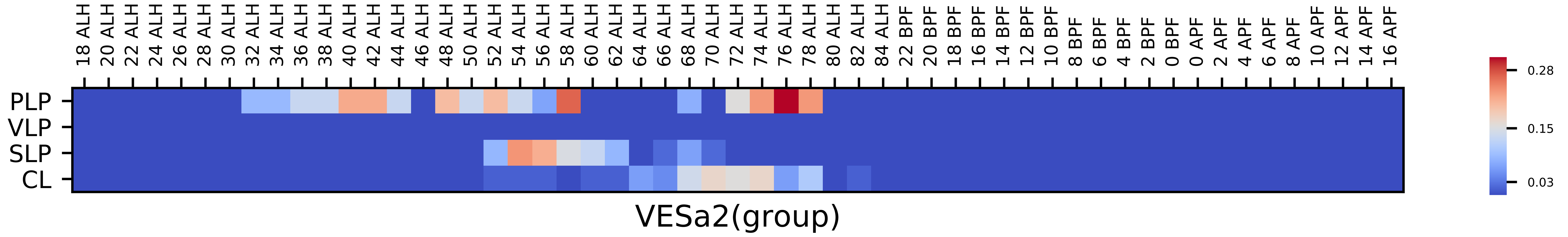

Figure 1-source data 1P-VLPa2

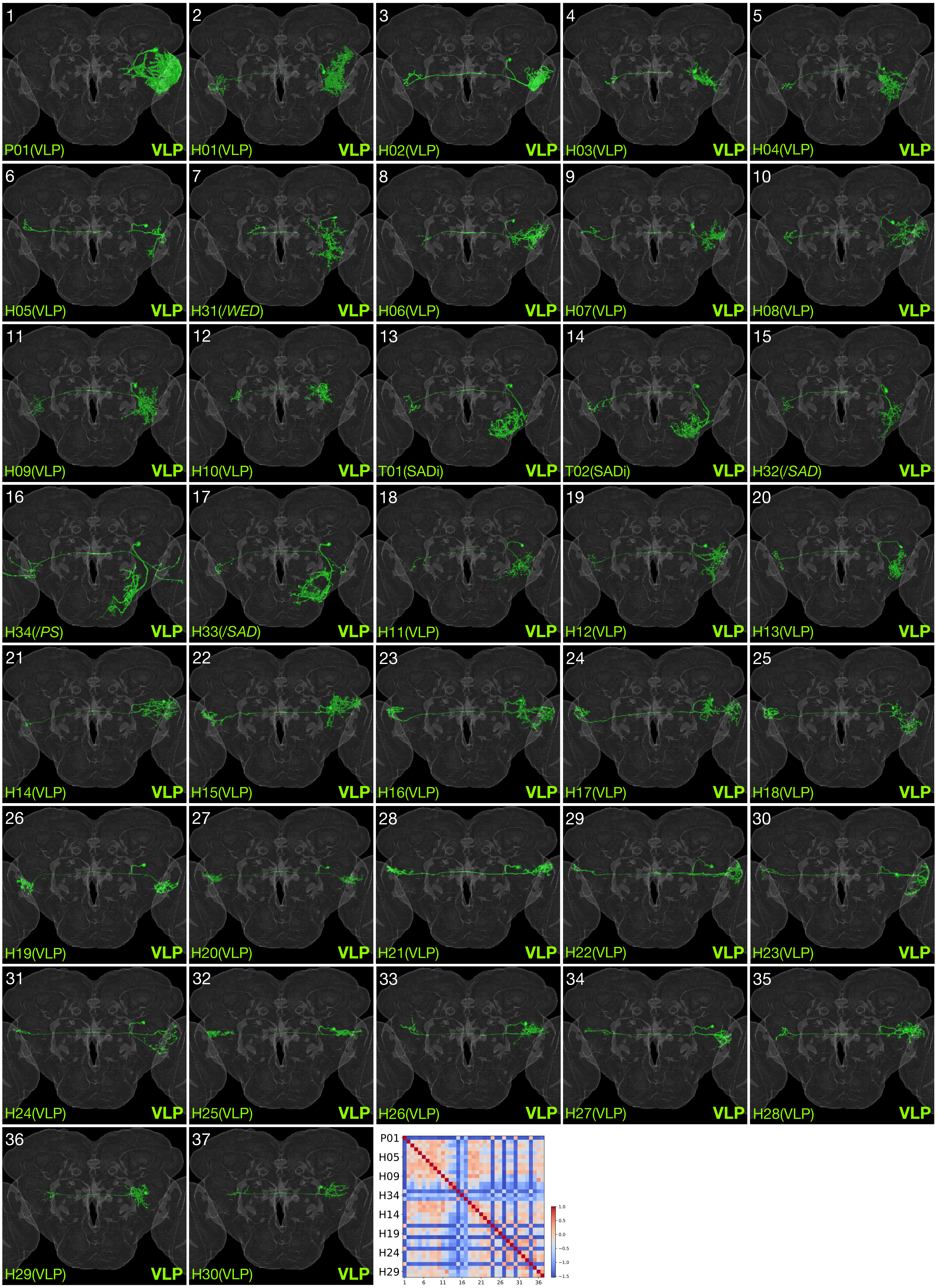

Figure 1-source data 1P-VLPa2-cont.

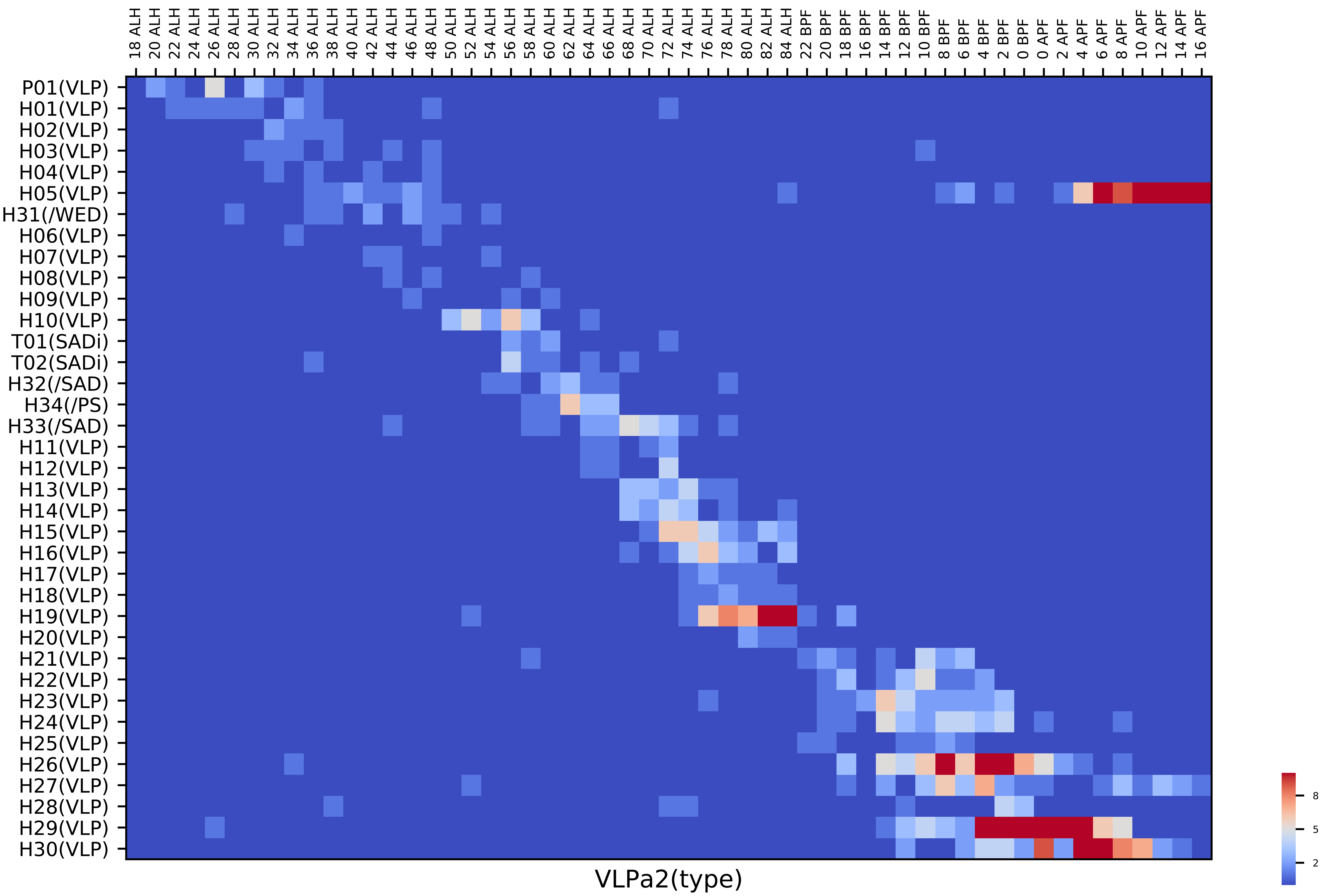

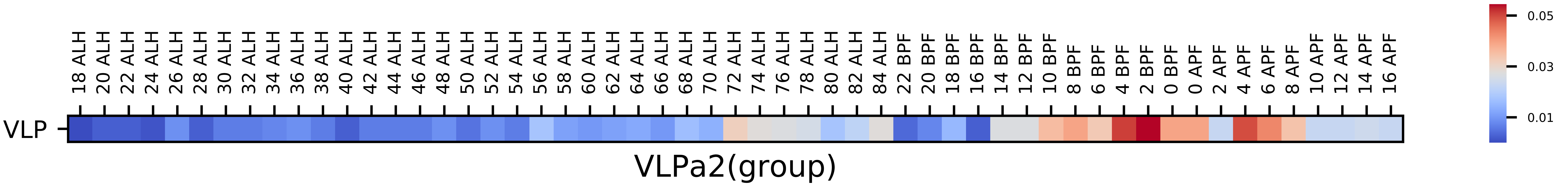

Figure 1-source data 1Q-WEDa1

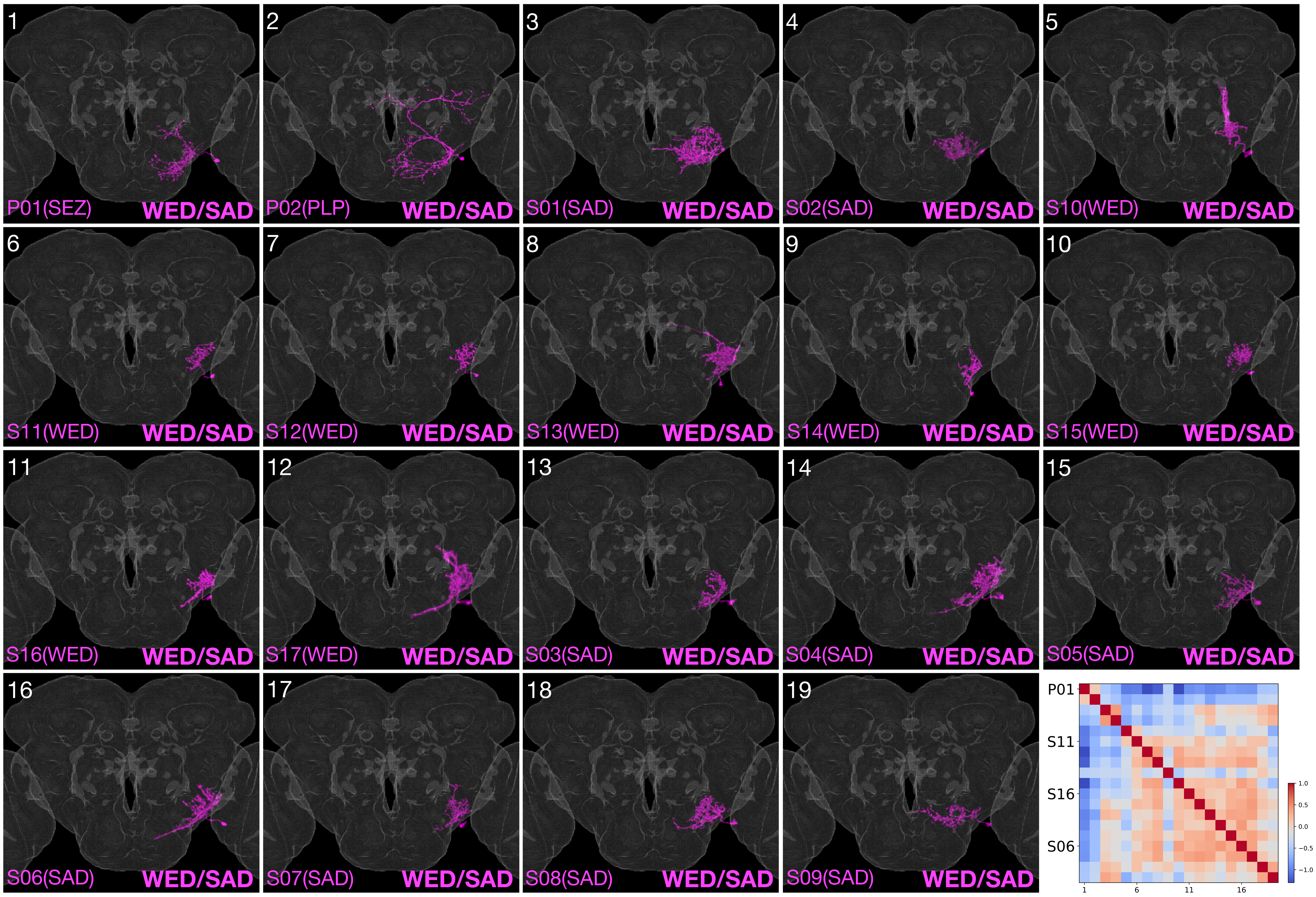

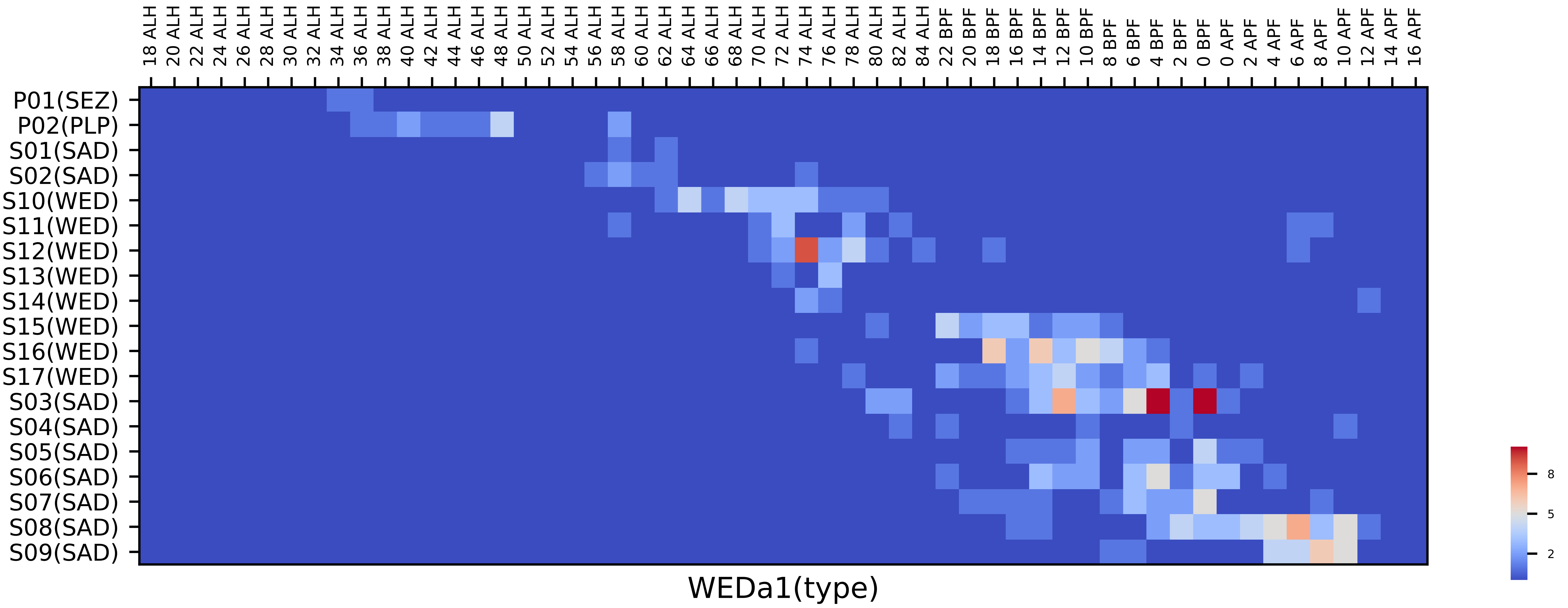

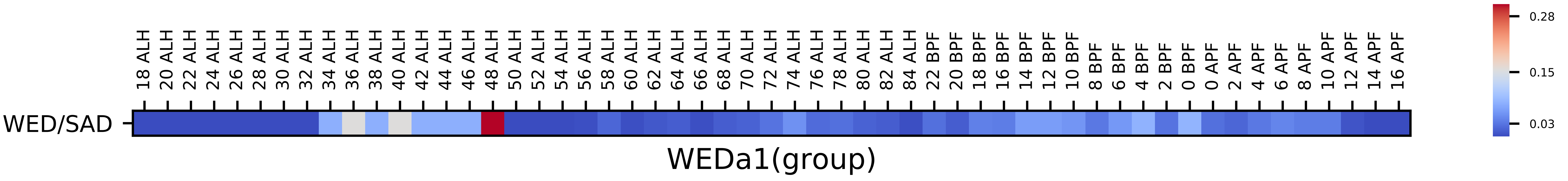

Figure 1-source data 1R-WEDd1

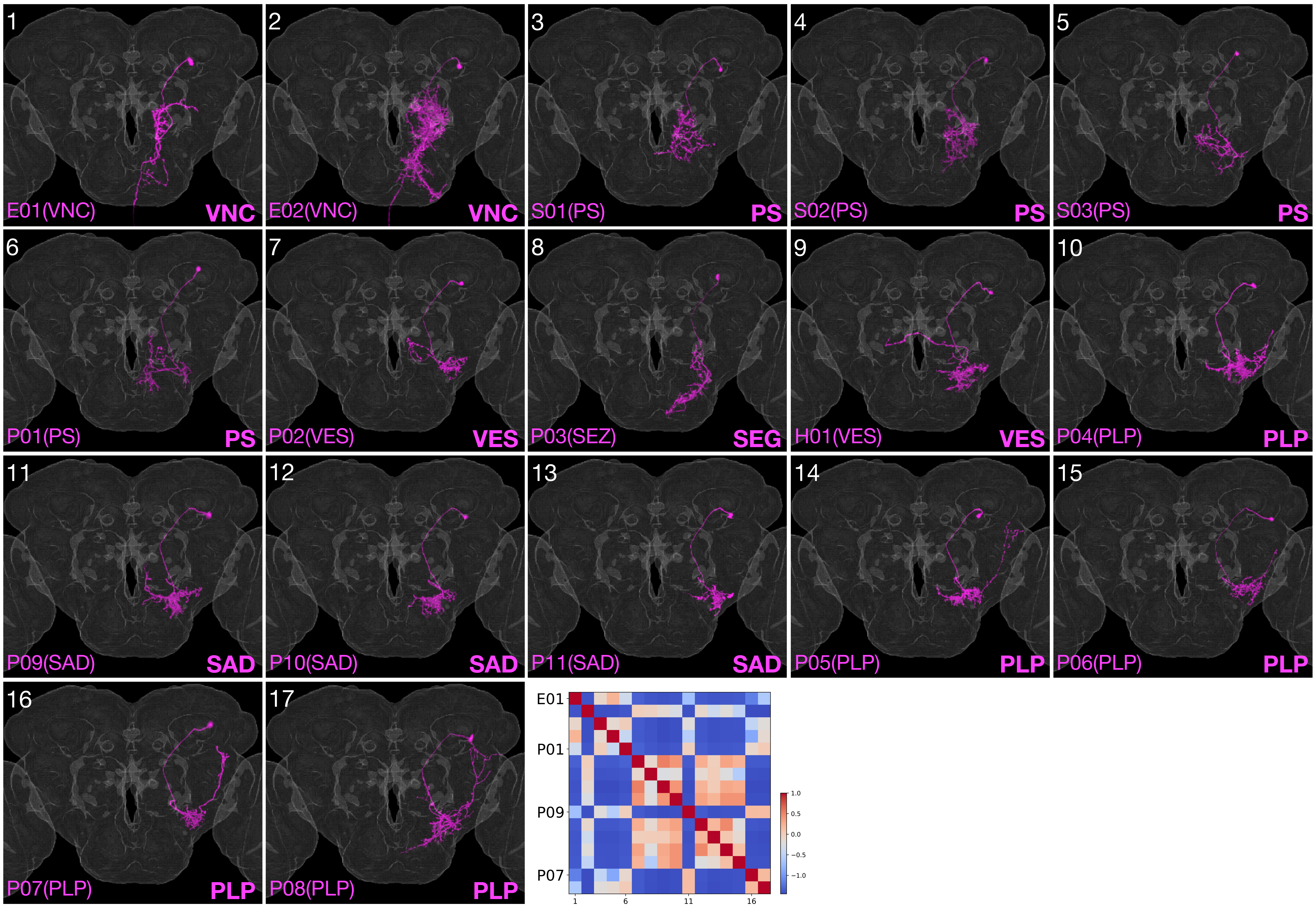

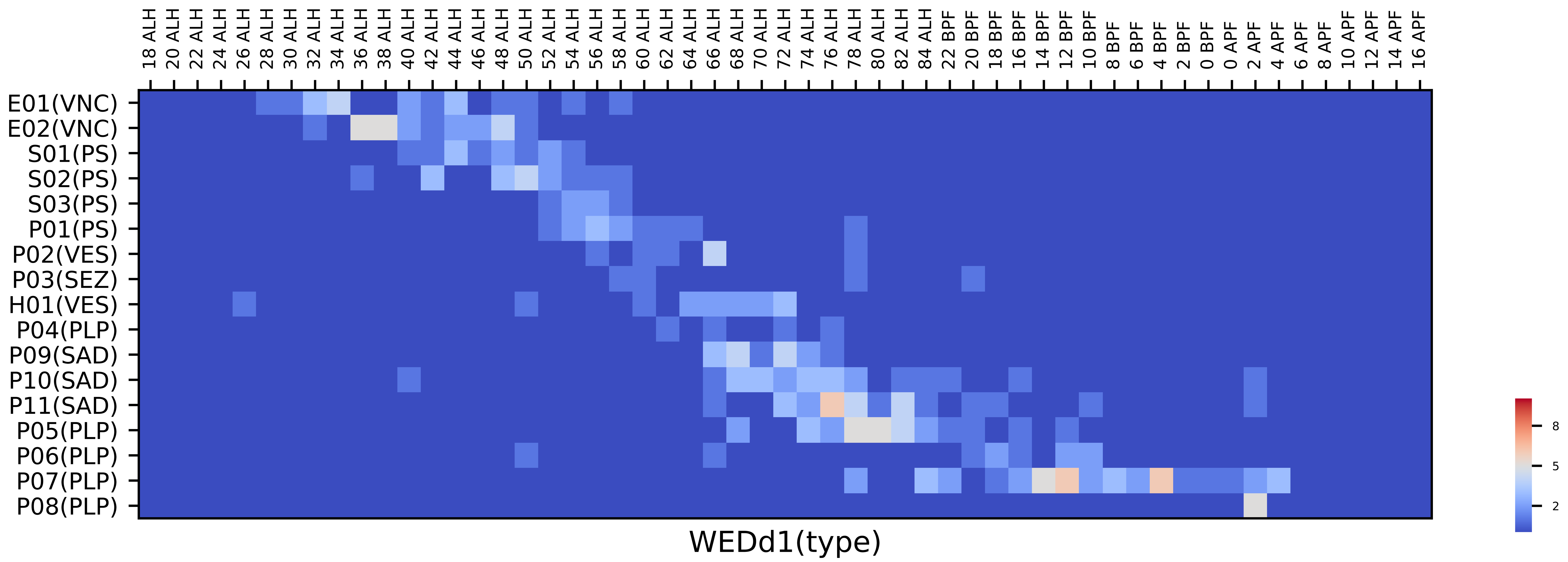

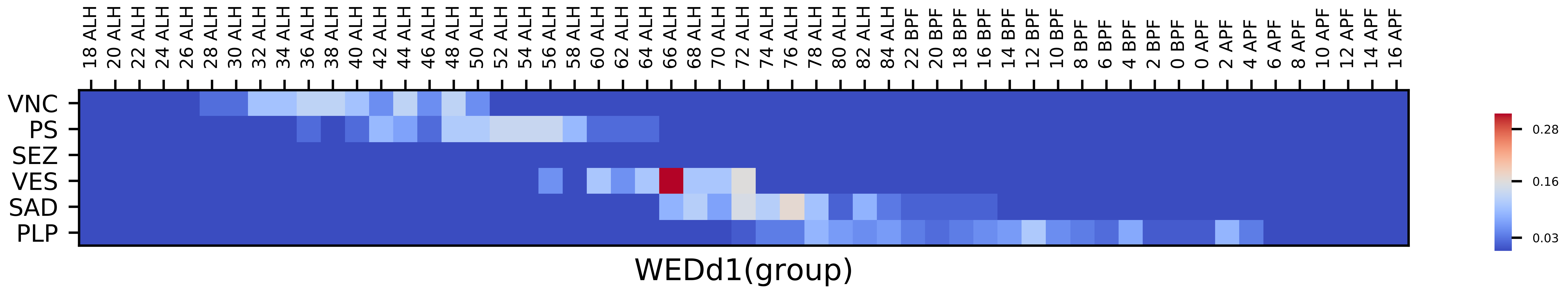

Supplement: Figure 1—source data 1. — Serially derived distinguishable twin-spot clones present in the same fly brain template (grey) for each of the 18 mapped Vnd lineages (shown in alphabetical order from ALv1 [A] to WEDd1 [R]). Note presence of either only one viable neuron or two sister neurons in GMC-derived twin-spot clones. Non: green; Noff: magenta. Within each hemilineage, we cluster neuron types (see text for neuron type nomenclature) into morphological groups named after main common neuropil targets (indicated on bottom right corner). The immediately following hemilineage-based heatmaps show NBLAST scores of pairwise neuron-type comparison, sorted based on the birth order. Other accompanying hemilineage-based heatmaps show orderly arrangement of neuron types or morphological groups on the Y axis based on their sequential recovery following clone induction at serial time points along the X axis (see Materials and methods for birth order analysis). For the type-level heatmaps, blue to red color represents the actual single-cell clone numbers (max = 10) recovered from induction at given time points. For the group-level heatmaps, the sample distribution was normalized to one for each separate production window. [file elife-53518-fig1-data1.pdf]
