# Supplementary figures and images for: Conservation and divergence of related neuronal lineages in the Drosophila central brain
Source: eLife. 2020 Apr 7;9:e53518. doi: 10.7554/eLife.53518 (PMC7173964; doi:10.7554/eLife.53518)

# Figure 1-source data 2

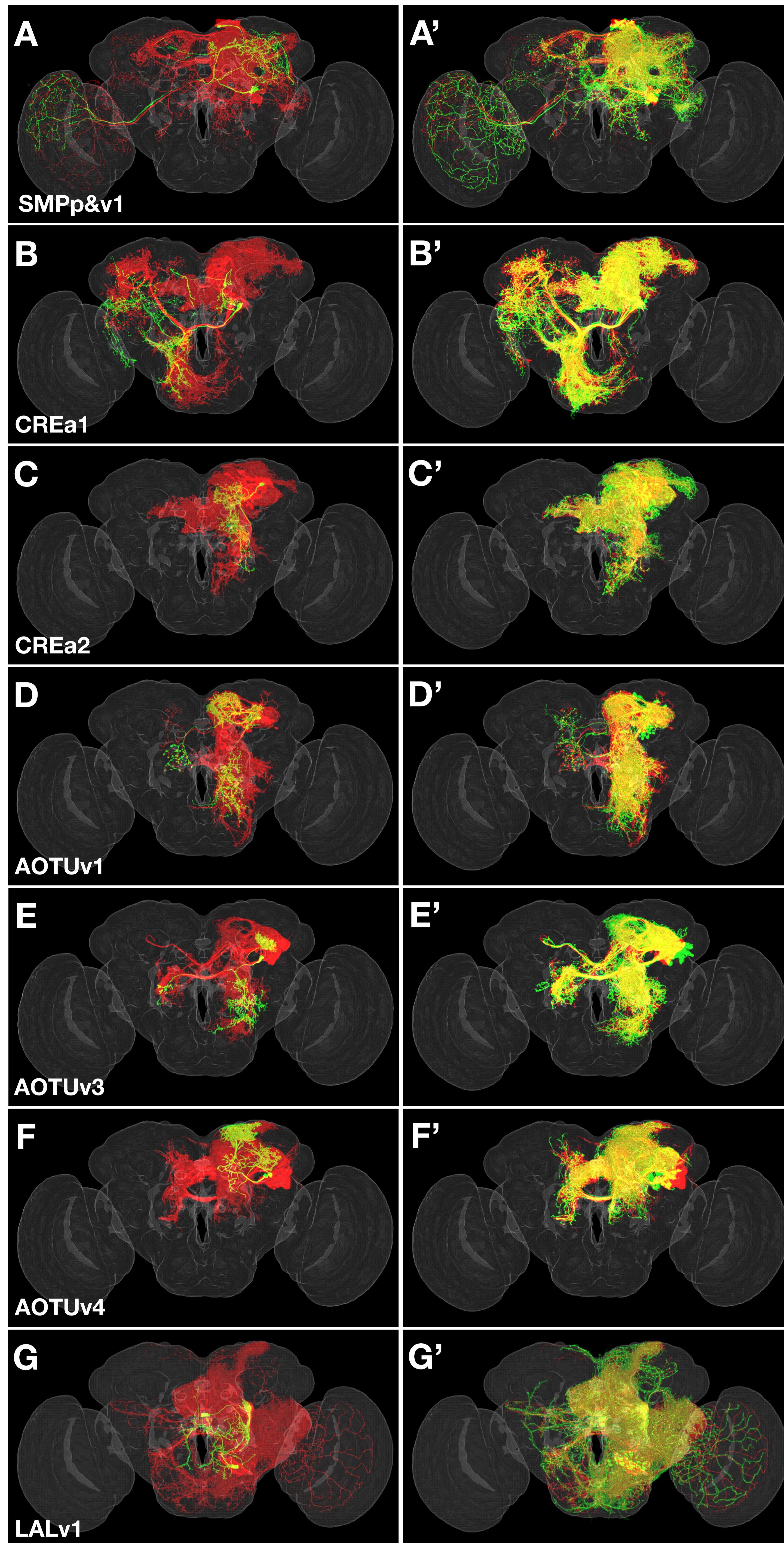

# Figure 1-source data 2

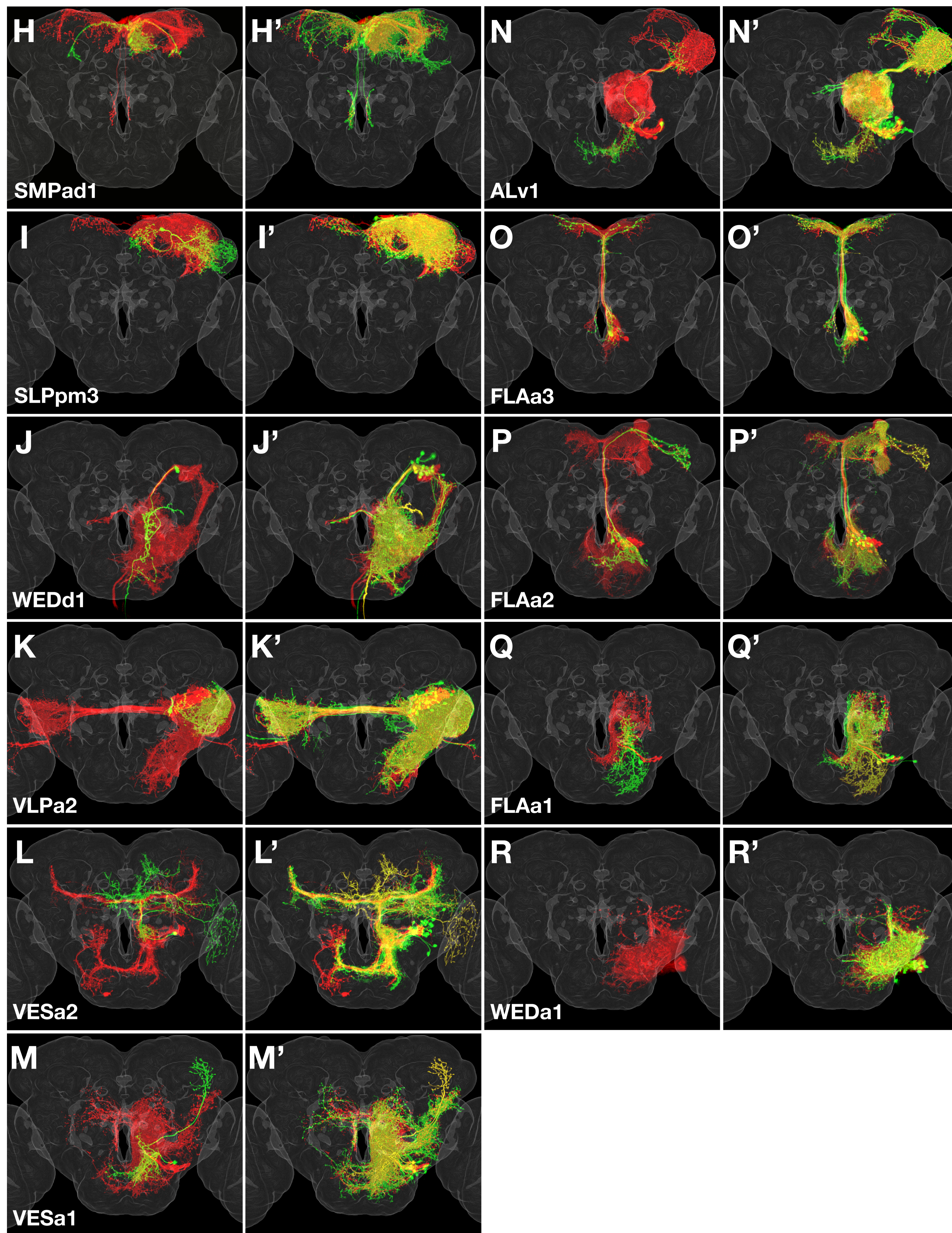

Supplement: Figure 1—source data 2. — (A–R) Representative twin-spot NB clones (GMC side: green, NB side: red) induced at the beginning of larval neurogenesis, shown in the standard fly brain template (grey). (A’–R’) For each of the 18 mapped Vnd lineages, we merged all morphologically distinguishable neuron types (green) for close comparison with the corresponding twin-spot NB clone. The twin spots of each twin-spot NB clone were pseudo-colored the same to derive the whole full-size pattern (red). To account for the entire pattern (red) with a minimal number of single neurons (green), we merged one neuron per type or one neuron per subtype for types with obvious variations. Note extensive overlap between the green and red patterns, except in [L’] where an unrelated SEZ single-cell clone existed coincidently with the VESa2 twin-spot NB clone. [file elife-53518-fig1-data2.pdf]
